# A Moderate Water Deficit Induces Profound Changes in the Proteome of Developing Maize Ovaries

**DOI:** 10.3390/biom14101239

**Published:** 2024-09-30

**Authors:** Thierry Balliau, Mariamawit Ashenafi, Mélisande Blein-Nicolas, Olivier Turc, Michel Zivy, Elodie Marchadier

**Affiliations:** 1 AgroParisTech, GQE—Le Moulon, PAPPSO, Université Paris-Saclay, INRAE, CNRS, 91190 Gif-sur-Yvette, France; thierry.balliau@inrae.fr (T.B.); mariamawit.ashenafi2011@gmail.com (M.A.); melisande.blein-nicolas@inrae.fr (M.B.-N.); michel.zivy@universite-paris-saclay.fr (M.Z.); 2 LEPSE, INRAE, Montpellier SupAgro, Université Montpellier, 34293 Montpellier, France; olivier.turc@wanadoo.fr

**Keywords:** *Zea mays*, water deficit, ovaries, proteome

## Abstract

Water deficit is a major cause of yield loss for maize (*Zea mays*), leading to ovary abortion when applied at flowering time. To help understand the mechanisms involved in this phenomenon, the proteome response to water deficit has been analysed in developing ovaries at the silk emergence stage and five days later. Differential analysis, abundance pattern clustering and co-expression networks were performed in order to draw a general picture of the proteome changes all along ovary development and under the effect of water deficit. The results show that even mild water deficit has a major impact on ovary proteome, but this impact is very different from a response to stress. A part of the changes can be related to a slowdown of ovary development, while another part cannot. In particular, ovaries submitted to water deficit show an increase in proteins involved in protein biosynthesis and in vesicle transport together with a decrease in proteins involved in amino acid metabolism and proteolysis. According to the functions of increased proteins, the changes may be linked to auxin, brassinosteroids and jasmonate signalling but not abscisic acid.

## 1. Introduction

Maize (*Zea mays* L.) is a major staple food crops and is expected to overtake wheat as the most widely grown crop by 2030 [1]. Mainly cultivated as a direct or indirect feed source through grain and by-products such as stover and silage, maize plays a crucial role in maintaining the food security for humans and livestock [2]. Maize is also used as a source for bioethanol production providing an alternative to fossil energy [2]. First domesticated in Mexico, maize has adapted from tropical to temperate environments over several millennia. Maize varieties are now widely cultivated throughout the world, including in northern countries, but remain particularly susceptible to water stress. Multiple climate models agree on an increase in the frequency and severity of periods of drought in the near future [3], and simulations of climate scenarios that combine hotter and drier conditions predict higher maize yield losses than climate warming alone [4].

In response to drought during vegetative growth, maize plants slow down their development and limit the growth of aerial parts [5]. Cell division and expansion are reduced [6], and stomatal closure reduces C0_2_ uptake [7] and slows photosynthetic activity [5]. On the contrary, root growth is favoured [8].

The reproductive phase is the most critical for maize. Water stress inhibits ovary growth, induces ovary abortion, and consequently reduces the number of kernels per ear [9]. Ovary abortion depends on both the ovary developmental stage at which the water deficit occurs [10] and the silk growth rate [11]. The higher sensitivity is particularly observed in the ear apical position containing the youngest ovaries (meristematic region) [5]. Ovary growth inhibition is not related to the loss of turgor classically observed in other water-stressed tissues [12]. Water deficit has been shown to delay silking by affecting the silk elongation rate in the same manner as leaf growth [13]. Silk expansion is driven by cell division and expansion, both being reduced as a consequence of water deficit [14]. Silking delay consequently increases the critical anthesis to silking interval (ASI), resulting in asynchrony between pollen and silk maturations and losses in yield that can reach more than 60% in drought-sensitive maize lines [15]. Several studies led to the identification of multiple QTLs for ASI [16,17,18,19], some of which interact with the watering condition, demonstrating the complexity of ASI genetic determinism.

Some gene such as *lo1*, *lo2*, *sp1*, *sp2*, *ig1* and *mac1* [20,21,22,23] were identified as implicated in meiosis and ovule development, but their involvement in water stress response has not been demonstrated. Metabolic pathways underlying ovules development has been investigated more. In normal conditions, sucrose reaches the ear by phloem transport, and ovary growth depends on this continuous C flow from the parent plant. Under drought conditions, carbohydrate and sugar supply to the ovary is inhibited due to photosynthesis inhibition [24,25]. This can be temporarily compensated by the conversion of ovary starch to glucose, while starch reserves are sufficient [26]. However, in the absence of a starch pool and the arrival of photosynthesis products, ovary development is aborted [25]. Consistent with the requirement of sucrose for ovary development [27], sucrose supply restores the development of ovaries [28,29], although invertase activity is only partially restored [25]. In severe water deficit, carbon metabolism in ovaries is strongly disrupted, and soluble sugar and starch contents are particularly affected [5]. Invertase activity (the hydrolysis of sucrose to glucose and fructose) is inhibited, sucrose concentration increases [12] while reducing sugars (such as glucose and fructose) and starch downstream products are depleted [25,28].

More recent studies [11,30] performed under moderate water deficit investigated its effect on ovaries on a more precise time scale. They showed that ovary abortion is indeed due to a slowing down of their development and a reduction in their expansive growth, which impair their silk emergence and thus their pollination. Changes in carbon metabolism occur after this reduction of growth and can rather be considered as consequences more than causes of ovary abortions.

Studies at the transcriptome level confirmed the results of the biochemical studies [11] and showed that under severe water stress, the genes encoding sucrose processing enzymes were down-regulated and some genes involved in senescence were up-regulated around pollination [31]. However, while transcriptomic studies offer the advantage of accessing fast and finely tuned molecular responses, they do not take into account post-transcriptional regulatory events which are known to strongly affect the metabolic composition of tissues [32,33]. As intermediary molecules, proteins offer interesting perspectives for a better understanding of molecular processes, providing information on post-transcriptional and post-translational regulatory events, the abundances of enzymes, and the active molecules that catalyse metabolic reactions. To go further in the understanding of the effect of water stress on ovary development, this study aims to better characterise their proteome under moderate water deficit.

## 2. Materials and Methods

### 2.1. Experimental Design

The reference line B73 was used in this experiment. B73 is a widely used inbred line in breeding, and it is the first maize inbred line having been sequenced [34]. Plants were grown in a greenhouse and watered up to tassel emergence. Irrigation was stopped for half the plants until they reached a water potential of ca. −0.4 to −0.6 MPa. All plants were then transferred to a growth chamber, where water deficit (WD) or well-watered (WW) conditions were maintained. Ears were manually pollinated daily from silk emergence. Ovary samples were collected at two stages: at silk emergence (SE) and 5 days after silk emergence (SE + 5d). At each stage, ovaries were collected from five zones of the ear (numbered 1 to 5 from bottom to top, i.e., from the oldest to the youngest ovaries), except for zone 5 of SE + 5d samples of WD plants because of ovary abortion at the top of the ear. Three biological replicates were taken for each condition × day × zone combination.

### 2.2. Proteomics

The extraction and digestion of ovary proteins were performed according to the liquid digestion protocol described in [35]. Briefly, proteins were precipitated in a TCA/acetone solution and then solubilised in a urea/thiourea solution containing dithiothreitol and zwitterionic acid labile surfactant. After alkylation with iodoacetamide, proteins were digested with trypsin and desalted on a C18 solid phase extraction column. Mass spectrometry (MS) analysis was performed by using an LTQ-Orbitrap coupled to an Ultimate 3000 LC system (Thermo, Waltham, MA, USA) as described in [36]. Protein identification and quantification were performed using i2MassChroq (version 0.6.0, release 15 April 2022, O. Langella, Gif-sur-Yvette, France http://pappso.inrae.fr/bioinfo/i2masschroq/, access 23 August 2024), which uses X!Tandem (X! Tandem Alanine (2017.2.1.4) [37]) for protein identification, XtandemPipeline [38] for protein inference, and MassChroQ (version 2.1.0) [39] for quantification. The genome assembly B73 RefGen_v4 database was searched (access 27 May 2020) https://ftp.ensemblgenomes.ebi.ac.uk/pub/plants/release-50/fasta/zea_mays/pep/Zea_mays.B73_RefGen_v4.pep.all.fa.gz. In total, 1815 proteins were identified with at least two peptides with an E-value < 0.05 and a protein E-value < 0.0001. The false discovery rate for peptide and protein identification was 0.27% and 0.42%, respectively. Peptide quantification was performed by the integration of extracted ion current (XIC). Normalisation was performed as described in [40]. Protein relative abundances were computed by summing the XIC value of specific and reproducible peptides (missing values < 10%), which showed a correlation to each other >0.5. Finally, a quantitative relative abundance was obtained for a total of 800 proteins.

### 2.3. Principal Component Analysis

Principal component analysis was performed on the entire dataset with the mean protein abundances as variables and the different condition × day × zone combinations as individuals. The prcomp function of the R package [41] was used.

### 2.4. Clustering Method

The 800 quantified proteins were grouped into eight clusters by using the K-means method on protein mean abundances computed for each condition × day × zone combination (kmeans function of the stats R package). Mapman annotations of proteins were collected by using Mercator 4 [42]. Independence between Mapman annotations and cluster assignments was tested using the independence Chi-squared test.

### 2.5. Differential Analysis

The following model was applied to the abundance variable of each of the quantified proteins, where $Y_{ijkl}$ represents the observed abundances, μ represents the mean abundance of the protein, $\alpha_{i}$ represents the condition *i* effect, $\beta_{j}$ represents the zone *j* effect, $\delta_{k}$ represents the day *k* effect, $\gamma_{ij}$, $\theta_{ik}$ and $\omega_{jk}$ represent the pairwise interactions of the effects, and $\epsilon_{ijkl}$ represents the residuals.

$$Y_{ijkl}∼\mu +\alpha_{i}+\beta_{j}+\delta_{k}+\gamma_{ij}+\theta_{ik}+\omega_{jk}+\epsilon_{ijkl}$$

Type III analysis of variance (ANOVA) was applied to this model (ANOVA function of the car R package), and *p* values were corrected using the Benjamini–Hochberg multiple testing correction procedure [43]. An effect was considered significant if the adjusted *p* value was less than 0.05.

### 2.6. Network-Based Analyses

#### 2.6.1. Identification of Protein–Protein Co-Expressions

For each combination of date, zone, and condition, the protein abundances of the three replicates were averaged. Then, Pearson correlation coefficients were then calculated for each pair of proteins for control and stress conditions independently, resulting in a correlation matrix per condition. P values were adjusted using the Benjamini–Hochberg correction for multiple testing, and correlations with adjusted *p* values less than 0.05 were kept as significant.

#### 2.6.2. Analysis of WW Network

The density of the WW network was calculated using $p_{WW}=\frac{n_{WW}}{800\times 799/2}$ where $n_{WW}$ refers to the number of co-expressed proteins in WW. Then, for each pair of Mapman terms, the proportion of expected protein–protein connections was calculated under the assumption that there were no preferential connections between Mapman terms. These theoretical proportions were compared with the observed numbers of connections between pairs of Mapman terms. Pairs of Mapman terms whose proteins were significantly more or less connected together were identified using a hypergeometric test.

#### 2.6.3. Comparison of WW and WD Networks

At the general scale, comparisons between WW and WD networks were made using estimates of the probabilities of observing an edge in each of the networks $p_{WW}=\frac{n_{WW}}{800\times 799/2}$ and $p_{WD}\frac{n_{WD}}{800\times 799/2}$ where $n_{WW}$ and $n_{WD}$, respectively, refer to the number of co-expressed proteins in WW and WD networks. Assuming the independence between the two networks, the probability of observing an edge in WW and WD networks is equal to $p_{WW}\times p_{WD}$.

On a more specific scale, for each pair of Mapman terms, we identified the number of connections involving both of these terms and the number of other connections in WW and in WD. Significant changes in connectivity between WW and WD networks were identified using Chi-squared tests of independence, and Benjamini–Hochberg [43] adjusted *p* values less than 0.05 were considered as significant.

### 2.7. KEGG Pathway Mapping

KEGG orthology (KO) compound identifiers corresponding to lists of differentially expressed proteins were obtained from the Phytozome website (https://data.jgi.doe.gov/refine-download/phytozome?q=zea&expanded=Phytozome-493, access 19 February 2024). KEGG pathways involving the KO compounds were obtained using the KEGG API links (https://rest.kegg.jp/link/ko/pathway and https://rest.kegg.jp/list/pathway, access 19 February 2024). Tables with two columns were created. In the first column, the KO compounds corresponding to each of the proteins were stored. In the second column, numerical values corresponding to the overexpression (+1) and underexpression (−1) of each protein were indicated. This information was mapped to selected pathways using the KEGG procedure available at https://www.genome.jp/kegg/mapper/, access 23 August 2024.

## 3. Results

To better understand the response of maize ovaries to a moderate water deficit, we compared the proteome of developing ovaries under WD and WW conditions at two sampling days and in different zones of the ear. As the meristematic zone is located at the top of the ear, the lowest zones represent the oldest ovaries and the highest represent the youngest.

### 3.1. Main Factors Explaining Changes in Protein Abundances

**Figure 1:**
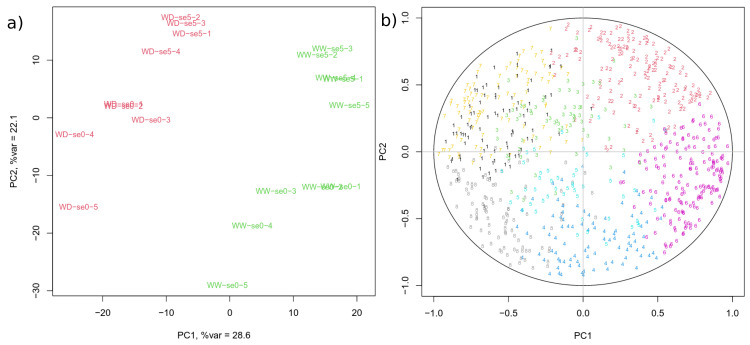
**Principal component analysis on ovary proteins abundances**. (**a**): Samples are named according to the concatenation of treatment, day and zone. se0 and se5 stand for SE and SE + 5d, respectively. Red: WD, green: WW. (**b**): Correlation circle. Proteins are represented by the number of the cluster to which they belong.

Proteome analysis using mass spectrometry allowed the quantification of 800 proteins (Table S1). To assess the effects of the condition, day, and zone factors on the proteome, principal component analysis (PCA) was performed (Figure 1). Principal components (PC) 1 and 2 represent 26.8% and 22.1% of the total variation, respectively. The PCA shows a strong condition effect: the WD and WW samples were clearly separated along the PC1 axis. A strong day effect is also evident, as SE + 5d samples are separated from SE samples within each condition along the second diagonal of the PC1/PC2 plane. The PCA also allows to distinguish the zones of the ear mainly at SE, where the samples are ordered according to the zone number, with zones 5 (youngest ovaries) and 4 separated from the group formed by zones 1, 2, and 3 along the first diagonal both for WD and WW conditions.

### 3.2. Main Functions Affected by Water Deficit and Development

**Figure 2:**
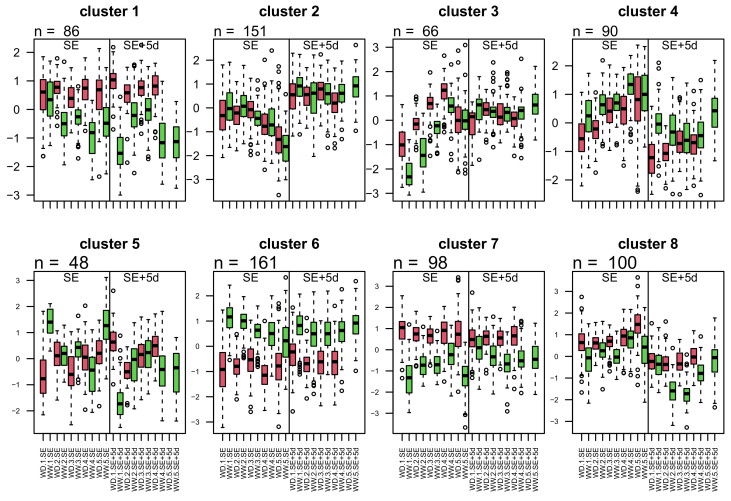
**Cluster profiles**. Each graph represents the abundance profile of the proteins making up a cluster in the different treatment*day*zone combinations. red: WD, green: WW.

To better characterise the patterns of protein abundance variation under the influence of WD and ovary development, a clustering analysis was conducted. This led to the identification of eight clusters of proteins, whose abundance patterns are shown in Figure 2 (Table S2). The functional categories of the proteins were not evenly distributed among the clusters (Chi-squared test *p* value $<2.2\times 10^{-16}$). The proteins belonging to the same cluster are clearly grouped together on the PCA correlation circle (Figure 1), which allowed us to define four classes of clusters according to the effects to which their proteins are subjected:

(i) Developmental effect: Cluster 2 shows a strong developmental effect. In this cluster, protein abundances decrease from the base to the top of the ear at SE, which is consistent with an increase in protein abundance during development, as the younger zones are higher up. For these proteins, the abundance would stabilise after this initial increase, as evidenced by the plateau observed at SE + 5d.

(ii) Primary condition effect: Clusters 1, 6, and 7 all contain proteins for which the response to WD is independent of the ovary developmental stage, since in the PCA, their proteins contribute maximally to the separation between conditions. For clusters 1 (86 proteins) and 7 (98 proteins), the median protein abundances in all day*zone combinations are higher in the WD condition than in the WW condition, whereas the opposite is observed for cluster 6 (161 proteins).

(iii) Secondary condition effect: For clusters 3, 4, and 8, a developmental effect (day and zone) predominates, but a WD effect is also visible. In cluster 3 (66 proteins), protein abundance increases from the bottom to the top of the ear at SE, while it is stable along the ear at SE + 5d. Proteins in cluster 4 (90 proteins) are mostly characterised by a higher abundance at SE than at SE + 5d, and there is a lower abundance in the WD condition than in the WW condition in the lower part of the ear at both stages. Cluster 8 proteins are more abundant at SE than at SE + 5d, and they are more abundant in the WD condition than in the WW condition at both stages.

(iv) No developmental or treatment effects: Cluster 5 is located at the center of the PCA correlation circle, indicating that it exhibits no variation based on any of the developmental or treatment factors.

**Figure 3:**
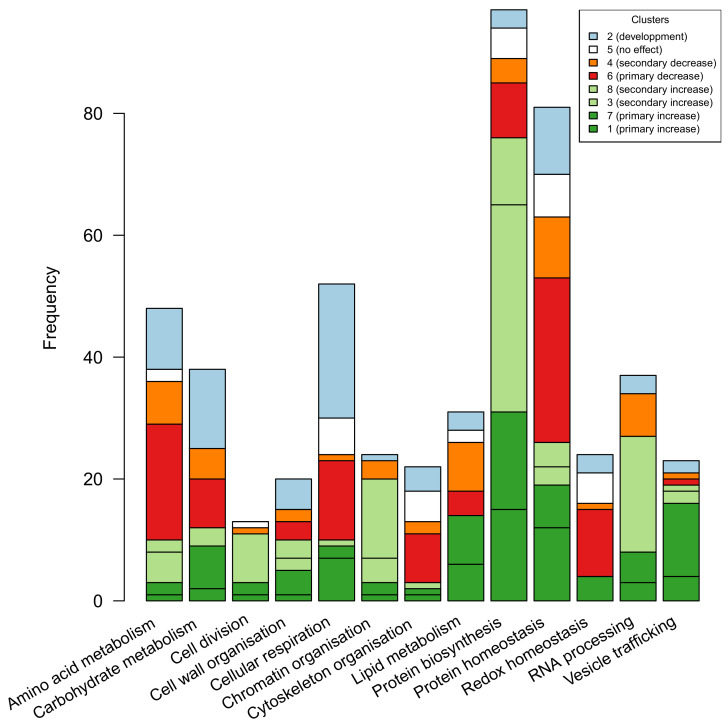
**Distribution of functional categories among clusters**. Red-scaled colors refer to proteins whose abundances decrease in WD, green-scaled colors refer to proteins whose abundances increase in WD, blue refers to proteins whose abundances change depending on ovary development. Only functional categories (Mapman level 1) represented by more than 20 proteins and the “Cell division” category are shown.

The major protein functional categories involved in these four cluster classes are shown in Figure 3. We focused particularly on the functional categories affected by WD and represented in classes (ii) and (iii). For four of them, most of the proteins showed a primary or a secondary increase in response to WD: protein biosynthesis (76 up-regulated proteins out of 97, mostly ribosomal proteins), RNA processing (27 up-regulated proteins out of 37), chromatin organisation (20 up-regulated proteins out of 24), vesicle trafficking (16 up-regulated proteins out of 23). For four other functional categories, most of the proteins showed a primary or a secondary decrease in response to WD: protein homeostasis (40 down-regulated proteins including 22 of the ubiquitin–proteasome system out of 81 proteins), amino acid metabolism (26 down-regulated proteins out of 48 proteins), redox homeostasis (12 down-regulated proteins out of 24), cytoskeleton organisation (10 down-regulated proteins out of 22). In the protein homeostasis category, chaperone proteins were equally distributed among up- and down-regulation (11 vs. 11 proteins). In the case of carbohydrate metabolism (38 proteins), 5 out of the 7 proteins involved in starch metabolism showed a primary increase in response to WD, and 4 out of the 11 proteins involved in sucrose metabolism showed a primary or secondary decrease. In the case of lipid metabolism (31 proteins) and cell wall organisation (20 proteins), 28 and 13 proteins, respectively, are affected by WD without showing a consensus response with respect to their function.

### 3.3. Pathways Affected by Water Deficit and Development: Differential Analysis

A differential analysis was performed considering the developmental (zone and day), condition, and interaction effects (Table S3 and Table 1). Boxplots representing the abundance patterns are shown in File S1 and the mapping of proteins to KEGG pathways is shown in File S2 and Table S6.

#### 3.3.1. Developmental Effects

To analyse the effect of ovary development on the proteome, we considered both the zone and the day effects. The differences observed between zones (162 proteins significantly affected) are informative regarding the short-term ovary development (from the oldest ovaries in zone 1 to the younger ones in zone 5), whereas those observed between days (327 proteins significantly affected) refer to a longer time scale (5 days). Among the proteins that are more expressed in young than in old ovaries, we found proteins involved in DNA replication (zone and day effect), proteins related to the spliceosome (zone effect), proteins involved in protein degradation outside the endoplasmic reticulum (day effect), proteins involved in starch and glucose metabolism acting toward starch synthesis (Zm00001d003817 for starch branching enzyme [EC:2.4.1.18] and Zm00001d019479 for granule-bound starch synthase [EC:2.4.1.242]; zone effect), and enzymes involving pyruvate as a substrate or a product (day effect). These notably include the enzymes allowing pyruvate interconversion from phosphoenolpyruvate, acetyl-CoA or malate (Zm00001d052494, Zm00001d001831, Zm00001d023379 and Zm00001d018961 for pyruvate kinase [EC:2.7.1.40]; Zm00001d006107, Zm00001d000227, Zm00001d052289 and Zm00001d004473 for pyruvate dehydrogenase [EC:1.2.4.1]; Zm00001d037961 for malic enzyme [EC:1.1.1.40]).

Among the proteins that are less expressed in young than in old ovaries, we found glycolysis proteins (zone and day effects), proteins of starch and glucose metabolism acting toward glucose synthesis (like Zm00001d023994, Zm00001d034015 and Zm00001d033649 for β-glucosidases [EC:3.2.1.21]; zone effect), proteins involved in citrate cycle and fructose–glucose interconversion (day effect), and proteins of the ER that are involved in protein processing and ribosome components (day effect). These proteins indicate that translation is more active in older ovaries.

Many proteins involved in the citrate cycle, pyruvate metabolism, and carbon fixation are affected in their abundance depending on the ear zone. However, no consistent pattern is observed between proteins of a common pathway. Amino acid metabolism also appears to be strongly affected by the day, but the direction of increased abundance varies greatly between reactions, which does not allow for identifying a consistent pattern.

The differential analysis identified binary interaction effects for some of the proteins. The interaction day*zone allowed us to identify 52 proteins, suggesting that the different zones develop differently over time. Among these proteins, we identified three chitinases (Zm00001d027524, Zm00001d025753, and Zm00001d026415), one of which is involved in the stress response of maize kernels [44]. Two proteins involved in α-linolenic acid metabolism were also identified (Zm00001d015852 and Zm00001d029594). This pathway is known to mediate the drought response in maize and leads to the biosynthesis of jasmonic acid [45]. In addition, numerous proteins involved in the ribosomes are identified with a significant day*zone interaction.

#### 3.3.2. Major Effect of Water Deficit

The differential analysis identified 481 proteins whose abundance was significantly affected by water deficit. Several of them belong to the same pathways, allowing a better understanding of the metabolic changes induced by WD. The proteins that are up-regulated under WD are mostly involved in chromatin organisation, RNA processing, spliceosome, protein processing, and vesicle trafficking, in particular proteins of the coatomer machinery (such as Zm00001d004466, Zm00001d007075, Zm00001d008218, Zm00001d016402, Zm00001d028438, and Zm00001d033734) in the WD condition. The proteins involved in RNA processing include a Tudor-SN protein (Zm00001d050606), which is involved in the formation of stress granules [46,47]. The proteins related to chromatin organisation include two members of the Argonaute family (Zm00001d039214 and Zm00001d040429) and a component of the SUVH-DNAJ methylation reader complex (Zm00001d038541).

Conversely, the down-regulated proteins are mainly involved in amino acid metabolism, protein homeostasis, carbohydrate metabolism, polyamine metabolism, redox homeostasis, and cytoskeleton organisation. Notably, three proteins involved in proline synthesis (Zm00001d010056, Zm00001d038689, and Zm00001d042117) are decreased, whereas one involved in proline catabolism (Zm00001d038841) is increased. Additionally, three proteins involved in GABA metabolism are significantly affected by WD: a γ-hydroxybutyrate dehydrogenase and a glyoxylate reductase (Zm00001d050810) are decreased, while a GABA pyruvate transaminase (Zm00001d002326) and a glutamate decarboxylase (Zm00001d031749) are increased. The proteins of redox homeostasis that are decreased are involved in ascorbate-based homeostasis (Zm00001d011034, Zm00001d024633, Zm00001d032950 and Zm00001d035595) and thiol-based homeostasis (Zm00001d011581, Zm00001d017823, Zm00001d040341 and Zm00001d046682). Therefore, it cannot be concluded that the proteome exhibits a response to oxidative stress. There are a few exceptions: the proteins that show a large increase are two 9-lipoxygenases (Zm00001d013493 and Zm00001d042540) that may be involved in brassinosteroid signalling [48].

Interestingly, two groups of proteins exist in carbon metabolism: proteins catalysing reactions leading to glucose which are down-regulated, and those leading to starch which are up-regulated in WD. The abundance of proteins involved in oxidative phosphorylation is also higher in WD, especially components of F-type mitochondrial ATP synthase and their related enzymes. On the contrary, some subunits of V-type ATP synthase (located in other endomembrane compartments [49]) are less expressed in WD. The effect of WD on carbohydrate and fatty acid metabolism appears to be more complex. Many enzymes involved in carbon metabolism, and especially pyruvate metabolism and the citrate cycle, are down-regulated, except for a few key enzymes. For instance, enzymes capable of interconverting oxaloacetate, citrate, and isocitrate (Zm00001d048358 and Zm00001d009127) are more highly expressed in WD. Among two enzymes able to convert oxaloacetate to citrate, one is down-regulated (Zm00001d053684, [EC:2.3.3.1]) whereas another one (Zm00001d009127, [EC:2.3.3.8]) is up-regulated in WD and particularly at SE. Enzymes that convert oxaloacetate to phosphoenolpyruvate such as phosphoenolpyruvate carboxylase PEPC (Zm00001d012702, Zm00001d016166, Zm00001d020057 [EC:4.1.1.31]) are also more highly expressed in WD. In addition, the enzyme catalysing phosphoenolpyruvate to aromatic amino acid precursors (Zm00001d006900) is up-regulated in WD at SE, whereas many enzymes involved in amino acid biosynthesis are down-regulated in WD. Enzymes involved in fatty acid synthesis and degradation and proteins involved in cell wall organisation also show significant abundance variations in response to WD. However, the proteins belonging to the same pathway do not exhibit similar patterns.

#### 3.3.3. Minor Effect of Water Deficit

The effect of the condition*day interaction is significant for 21 proteins, six of which also show a significant day*zone interaction effect. Three of them are enzymes of the starch and glucose metabolism and have glucose and starch as direct products or substrates. Two proteins exhibiting differential abundance are expansins (Zm00001d045861 and Zm00001d034663), which is a family of proteins involved in cell wall loosening and in mitigating the effects of drought on maize yield [50]. Their abundance profiles reveal their higher abundance in the WD condition at SE and their lower abundance in WD at SE + 5d (File S1). Only two proteins showed a zone*condition interaction effect, suggesting that the effect of the condition is not very different depending on the zone: Zm00001d027524, the chitinase mentioned above [44] and Zm00001d048021, a protein involved in α-linolenic acid metabolism (hydroperoxide dehydratase [EC:4.2.1.92]).

### 3.4. Changes between Protein–Protein Co-Expression Networks

To better understand how WD influenced protein abundance and to what extent new regulatory networks were set up, we compared the correlation of expression networks performed independently in WW and WD conditions (Tables S4 and S5). Within each condition, the only biological factors that vary are ear zone and sampling date, the combination of which can be used as a proxy for developmental stage. Thus, we compared the two co-expression networks obtained in each condition to better understand how WD affected the coordinated processes required for ear development.

Based on a significant correlation (BH-adjusted *p* values < 0.05), the WW and WD protein co-expression networks included 7824 and 2768 edges, respectively (Table 2). The number of common edges between both of the networks is much higher than expected by chance (624 in common versus 68 expected under the independence assumption of the networks-*p* value $<2.2\times 10^{-16}$), showing common protein abundance coordination through the ear developmental process in both of the watering conditions.

Table 2 also indicates that nearly 70% of the edges identified in the protein–protein co-expression networks involve at least one protein that was not identified by the differential expression analysis.

To analyse the biological significance of these networks, we used level 1 of the Mapman classification to group proteins involved in the same major process. Although each of these major processes can be made up of different pathways and can include different numbers of proteins, some general trends emerge.

#### 3.4.1. WW Network

**Figure 4:**
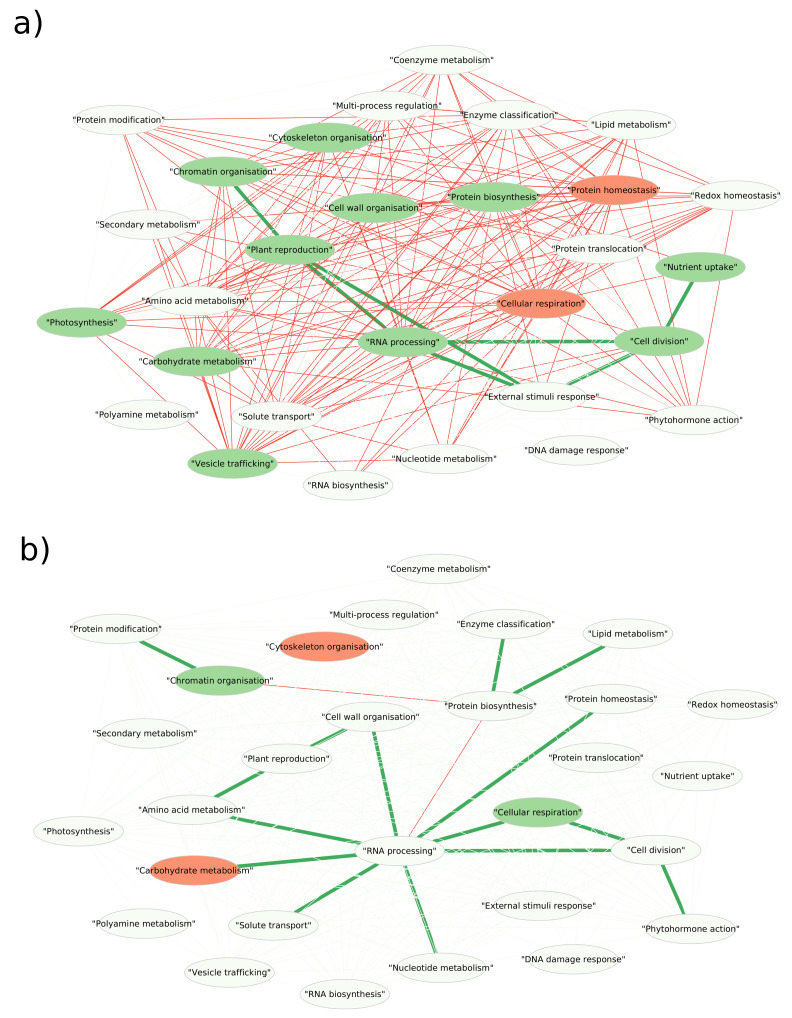
**WW network and differences between WW and WD networks**. (**a**) WW network: Mapman processes are represented by nodes. A green node represents a process for which the number of pairs of proteins co-expressed is significantly higher than expected by chance while a red node represents a process for which the number of pairs of proteins co-expressed is significantly lower than expected by chance. Similarly, green edges connect processes between which many pairs of proteins are co-expressed and red edges connect processes between which only a few pairs of proteins are co-expressed. (**b**) WD vs. WW network: Mapman processes are represented by nodes. Only pairs of Mapman terms whose proportions of connections differ between WD and WW were identified on this WD network. A green node represents a process for which the number of pairs of proteins co-expressed is significantly higher than in the WW network, while a red node represents a process for which the number of pairs of proteins co-expressed is significantly lower than in the WW network. Similarly, green edges connect processes between which many pairs of proteins are co-expressed compared to the WW network, and red edges connect processes between which only a few pairs of proteins are co-expressed compared to the WW network.

In a general manner, the network related to the WW condition (Figure 4) is rich in intra-process connections and low in inter-processes connections.

As shown in Figure 4, a majority of the Mapman processes have significantly more intra-process correlations than expected by chance. There are two exceptions: “protein homeostasis” and “cellular respiration” show less intra-process correlation than expected.

External stimuli response, nutrient uptake, cell division, plant reproduction, and transcription-related processes are identified as coordinated processes. The absence of connection with other processes for ”vesicle trafficking”, “protein biosynthesis”, “carbohydrate metabolism”, “photosynthesis”, “cytoskeleton” and “cell wall organisation” suggests that these processes independently participate in ovary development in the WW condition.

#### 3.4.2. Effect of WD on the Network

The network related to the WD condition was compared to the control network, and significant changes in intra and inter-processes connectivities were highlighted (Figure 4). Compared to the WW network, strong losses of intra-process correlations are observed for almost all processes, especially for “carbohydrate metabolism” and “cytoskeleton organisation”. Only “cellular respiration” shows a significant excess of intra-process correlations, while there were significantly fewer in the WW condition.

This global loss of intra-process connections is to the benefit of abundance correlations linking proteins of different processes. Three groups of connected processes appear to be independent from each other: (i) a large group of 11 interconnected processes, in which “RNA processing” occupies a central place since it is directly connected to 8 of the others. Among the latter, “cell division”, “phytohormones action” and “cellular respiration” form a subgroup of coordinated processes; (ii) “protein biosynthesis”, “lipid metabolism”, and “enzymes” and (iii) “protein modification” and “chromatin organisation”.

In the WD condition, the plant reproduction process (including three out of four proteins are involved in floral induction) appears coordinated with cell wall organisation and amino acid metabolism processes rather than with transcription-related processes as observed in the WW condition.

#### 3.4.3. Proteins Involved in the WW/WD Network Changes

The previous analysis showed that there were profound changes in the co-expression networks between the WD and WW conditions. Some changes are due to losses of connections, and some are due to gains. To better understand these changes, we analysed these gains and losses at the level of the proteins themselves.

**Figure 5:**
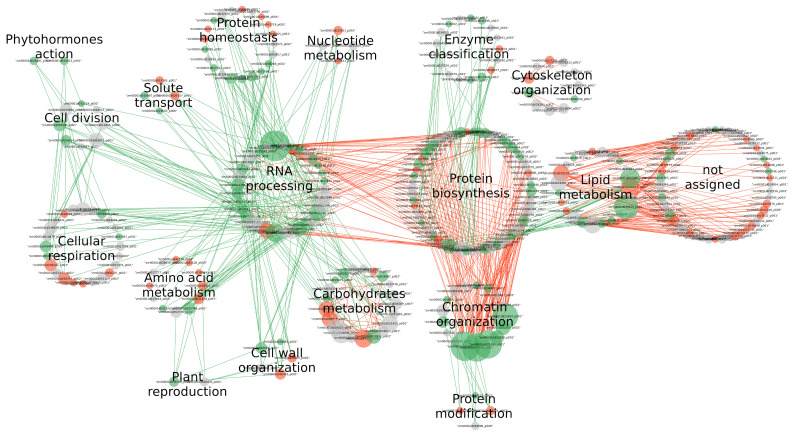
**“Water deficit” protein–protein network**. Nodes represent proteins involved in changes of inter and intra-Mapman processes connections. Node colors refer to the effect of WD on protein abundance (green: highly expressed in WD, red: less expressed in WD, white: not differentially expressed in WD), and node sizes refer to node degrees. Green edges represent correlations of protein abundances that are not observed in WW but are observed in WD, while red edges represent correlations of protein abundances that are observed in WW but lost in WD.

Figure 5 shows that several connections were lost due to correlations involving ’hub’ proteins whose abundances correlated with several other proteins in the WW condition. It is notably the case for the “RNA processing”, “lipid metabolism”, and ”chromatin organisation” processes. On the contrary, WD-specific correlations are more widespread among proteins of each of the processes (Figure 4 and Figure 5 and Table S7).

The most striking case is for protein Zm00001d006725, a translational terminator from the “RNA processing” process, which loses 31 connections to proteins from the “protein biosynthesis” process in the WD condition. Histone proteins from “chromatin organisation” also lose correlations of abundances with proteins involved in the “protein biosynthesis” process in the WD condition.

Several proteins involved in α-linoleic acid are co-expressed with diverse (“not assigned”) proteins in WW and lose these connections in the WD condition, except for “Zm00001d049059”, an alcohol dehydrogenase that loses co-expression with proteins associated with carbohydrate degradation (Zm00001d034256, Zm00001d035925 and Zm00001d049187) and sucrose synthesis (Zm00001d010523) to the benefit of co-expression with several proteins involved in “RNA processing”. A similar change of co-expression partners is observed for the sucrose synthase Zm00001d047253 protein, which lost co-expression with other proteins involved in sucrose metabolism (such as Zm00001d045042, Zm00001d029087, and Zm00001d010523) observed in the WW condition and gained several associations with “RNA processing” proteins in the WD condition.

## 4. Discussion

This study investigated the proteome response of B73 developing ovaries to a moderate water deficit that is sufficient to induce ovary abortion. Due to its great yield potential, B73 is a widely used inbred line in breeding and the first maize inbred line having been sequenced [34]. This line is sensitive to water stress during its vegetative growth [51] and during its reproductive phase, showing a strong yield reduction in water stress [52]. The effect of water deficit was analysed in the proteome of ovaries collected along the ear at the stage of silk emergence and five days later. In addition to the differential expression analysis, a clustering procedure allowed us to group together proteins with similar abundance profiles and protein abundance correlation networks, allowing to study the effect of water deficit on the functional coordination of developing ovaries.

The different approaches used in this study (PCA, ANOVA, cluster analysis, co-expression networks) showed that the effect of water deficit differs according to the stage of ovary development. As a first step, we will analyse the effect of development on the ovary proteome independently of water deficit.

### 4.1. Functions Involved in Ovary Development in the WW Condition

Our experimental design allowed to compare ovary developmental stages based on two factors: the zone of the ear, which allowed us to study early and short-term developmental processes, and the day of the sampling which allowed us to compare longer developmental time intervals. The results of the PCA show that samples from the different zones collected at silking emergence are more distinct than those collected five days later, suggesting that the gradient of ovary development along the ear is greater at silking emergence than five days later: more differentiation takes place during early stages of ovary development.

Previous studies of maize ovary development have highlighted the importance of carbon flow from the phloem and the conversion of sucrose to glucose, fructose and starch in this process [5,12,28]. In our study, the clustering and differential analysis approaches show that starch synthesis, as well as DNA replication, and transcription-related functions, are enriched in younger ovaries. Proteins involved in cell division are at higher levels in SE than in SE + 5d, and more than half of them show a strong ascending gradient in SE, which is consistent with the fact that cell division is more active in young cells. The protein degradation function is also highly expressed at silk emergence. Enzymes that allow the interconversion of metabolic hubs such as pyruvate, phosphoenolpyruvate, and acetyl-CoA are also more abundant at the silk emergence stage.

On the contrary, translation is more active in older cells, where ribosomal proteins are more abundant. Carbohydrate metabolism pathways with glycolysis and citrate cycle enzymes are also up-regulated in older ovaries. Precursors of jasmonic acid involved in plant development were also identified as affected during ovary development. Interestingly, the differential analysis identified an interaction between the day and zone effects for three chitinases whose abundances were higher in older ovaries (SE + 5d), which could correspond to a defence process against fungi that is established during ovary development.

In summary, the following changes have been shown to occur during ovary development:

- During the early stages of ovary development, DNA replication, transcription and protein degradation are active processes, while translation is reduced. The synthesis of starch and metabolic hubs is also favoured.
- Ovary maturation coincides with the activation of glucose metabolism, starch to glucose conversion and glucose catabolism by glycolysis are activated.
- Mature ovaries have active translation-related functions.

### 4.2. Response to Water Deficit

Although the plants were subjected to a moderate water deficit, our results show that it was the primary cause of protein variation: (i) the PCA clearly separated WD from WW samples along the first principal component; (ii) the highest number of significant differences identified by ANOVA was for the WD effect, (iii) the majority of the clusters showed a water deficit effect with or without interaction with a developmental effect. In addition, a large difference was observed between the WW and WD co-expression networks.

Irrespective of the developmental stage, ovaries in the WD condition are characterised by a higher abundance of starch synthesis proteins and enzymes catalysing interconversions between metabolic hubs and a lower abundance of proteins involved in carbon metabolism. This metabolic pattern is similar to that observed in young ovaries in the WW condition, suggesting that ovaries under WD behave like young ovaries and slow down their carbohydrate metabolism. WD is also associated with an increase in proteins involved in cell division, chromatin organisation, and RNA processing.

Consistently, the WD network highlights a loss of coordination between proteins involved in carbohydrate metabolism but also new connections between pathways in WD, while those observed in WW are still present, suggesting more coordination mechanisms between pathways in WD than in WW. The coordination between pathways might be based on RNA processing proteins.

This suggests that these proteins have a dominant regulatory role on different pathways under WD, whereas their role would be less important under the WW condition. Interestingly, some of them are indeed involved in the stress response. In particular, a Tudor-SN protein, which is known to be induced by stress, is primarily increased in the WD condition. It plays a role in sequestering mRNA in stress granules to allow the translation of stress-specific mRNA [46,47]. Enzymes catalysing the synthesis and degradation of metabolic hubs such as pyruvate, citrate, oxaloacetate, and phosphoenolpyruvate are abundant in the WD condition and could also explain the coordination of pathways. Analysis of protein co-expression networks identified alcohol dehydrogenase and sucrose synthase [53] as potential orchestrators of changes in the correlation of abundances.

Protein biosynthesis/processing pathways and vesicular trafficking are also activated in WD, whereas most enzymes of amino acid metabolism and proteins involved in protein degradation (mainly proteasome subunits) are decreased. In this respect, WD ovaries seem to follow their maturation process by maintaining their protein pool by activating translation and protein processing (ER) and limiting protein degradation. These processes do not seem to depend on amino acid biosynthesis. We can speculate that the increase in proteins involved in vesicular trafficking allows the remobilisation of intra- or extracellular protein material to be used as an amino acid supply. In association with this increase in vesicle trafficking proteins, two phospholipases D are induced in WD. These proteins play a role in stress signalling and vesicle trafficking [54].

Only two proteins with a strong condition*zone interaction effect were identified, whereas a significant effect of the day*condition interaction was observed for 21 proteins. This suggests that WD only slightly affects the short-term developmental program of the ovary, but it does so on a larger developmental time scale. As recently shown in leaves [55], the molecular response to water deficit is at least partly dependent on the age of the tissue. Indeed, as shown in cluster 3, some protein abundances are specifically affected by WD at the time of silk emergence. Among them, two expansins (proteins involved in cell expansion) appear to be induced by WD at the time of silk emergence, while their abundance decreases with WD at later stages of ovary development.

The moderate deficit applied in this study does not induce a typical stress response in the maize ovary proteome. There is no increase in proteins involved in the synthesis of osmoprotectants such as proline (pyrroline-5-carboxylate reductase) or betaine-glycine (betaine-aldehyde dehydrogenase), no general increase in chaperone proteins or dehydrins, and no increase in proteins involved in the defence against protein oxidative stress, either ascorbate or thiol-based in WD. In fact, although WD globally promotes oxidative phosphorylation that generates ROS, redox homeostasis is down-regulated.

Although there is no typical stress response, the ovary proteome is strongly affected by water deficit. Profound changes in the proteome are already observed at the stage of silk emergence.

Several hormone signalling genes were found to be up-regulated in response to abiotic stress in ovaries under heat stress [56]). Jasmonic acid and abscisic acid signalling are induced by drought in maize ears [57]. Auxins are involved in growth but also in in seed development [58,59] and response to drought stress [60]. Brassinosteroids interact with auxins to promote cell expansion [61] and play a role in drought stress and senescence [62]. Because of its importance in the response to environmental stresses such as water limitation, changes in abscisic acid (ABA) concentrations in drought-stressed ovaries have been investigated in several studies but with different results depending on the tissue. The ABA concentration per ovary fresh weight or per kernel increases [63], while it decreases when measured on the floret [64] and is stable when measured on the ovary dry weight [65]. In the present study, the observed changes do not seem to be related to ABA, as the abundance of proteins involved in its synthesis decreases after WD. On the other hand, other proteins involved in hormonal regulation increase in WD, such as a topless-related2 (TPL/TPR) protein with relevance to auxins. This protein and nana2, another protein increased in WD, may also be involved in brassinosteroid metabolism [66,67]. Two 9-lipoxygenases involved in brassinosteroid signalling [48] are also increased in WD. An acyl-CoA binding protein (ACBP6), a protein involved in jasmonic acid synthesis, is increased in WD. Taken together, our results suggest that the changes induced by water deficit are more related to auxins, brassinosteroids and jasmonate than to abscisic acid signalling.

Several responses to WD are typical of young ovaries. In particular, the abundance of proteins involved in transcription and glucose/starch balance is similar to that of young ovaries, suggesting that after WD, ovaries slow down their development and some proteins remain at the level they reached when young. Consistent with our results, the time course transcriptome analysis of ears under increased water stress showed an initial down-regulation of genes related to cell division (DNA replication and cell cycle), which is followed by a down-regulation of genes related to glucose metabolism, glycolysis and the tricarboxylic acid cycle [57].

Our results also support the conclusions of Oury et al. [11] showing that under water deficit, the down-regulation of carbon metabolism is not involved in the primary response of ears and that expansive growth is initially affected by WD. However, similar to older ovaries, translation is active in WD, but the amino acid pool could be supplied by alternative pathways than amino acid synthesis. Such changes in protein synthesis in response to WD have not been previously described.

## 5. Conclusions

Several conclusions can be drawn from this study. The proteome of the ovary changes during development with proteins involved in cell division and expansion being more abundant in young than in older ovaries. One of the changes induced by mild water deficit is a slowing down of development: in the WD condition, the abundance of several proteins resembles their level in younger ovaries.

But other changes cannot be linked to a slowdown in development: for example, proteins involved in protein biosynthesis are increased during water deficit. At the same time, proteins involved in amino acid metabolism and proteolysis decrease, raising questions about the availability of amino acids for translation. There is a concomitant increase in proteins involved in vesicle trafficking. The co-expression networks in WW and WD are very different, confirming that the changes are not only related to the developmental slowdown. These changes may be directly or indirectly regulated by several proteins involved in RNA processing, which are increased in WD. Even mild water deficit has a large and rapid effect on the ovarian proteome, but the response is different from a response to stress: the proteome does not show the standard symptoms of a response to water deficit, such as increased abundance of dehydrins, chaperones or proteins involved in redox homeostasis. The changes may be related to hormone signalling: several proteins involved in auxin, brassinosteroids and jasmonate, but not ABA, were up-regulated in response to water deficit.

## Figures and Tables

**Table 1:** Main functional categories for which protein abundance is impacted by developmental and/or condition effects.

Functional Category	Condition	Development Stage	Interaction
DNA replication	-	Young > Old	-
Chromatin organisation	WD > WW	-	-
RNA processing–spliceosome	WD > WW	Young > Old	-
Cell division	WD > WW	Young > Old	-
Cell growth	-	-	Up in WD Young Down in WD Old
Amino acid biosynthesis	WW > WD	-	-
Protein synthesis/ribosomes	WD > WW	Old > Young	-
Protein processing	WD > WW	-	-
Vesicule trafficking	WD > WW	-	-
Protein homeostasis/degradation	WW > WD	Young > Old	-
Oxidative phosphorylation	WD > WW	-	-
Redox homeostasis	WW > WD	-	-
Glucose–starch toward starch synthesis	WD > WW	Young > Old	-
Glucose–starch toward glucose synthesis	WW > WD	Old > Young	-
Glycolysis	-	Old > Young	-
Fructose–glucose interconversion	-	Old > Young	-
Pyruvate metabolism	WW > WD	Old > Young	-
Pyruvate conversion	-	Young > Old	-
Polyamine and GABA metabolism	WW > WD	-	-
Cytoskeleton organisation	WW > WD	-	-
Chitinases	-	Old > Young	Down in WD Old

**Table 2:** Number of correlations of protein abundances involving 0, 1 or 2 differentially expressed proteins (in columns) per network (in lines).

	No DEP	One DEP	Two DEP	Total
Common correlation network (WW and WD)	188	289	147	624
WW correlation network	1584	3837	2403	7824
WD correlation network	638	1341	789	2768
Total	2034	4889	3045	9968

## Data Availability

The mass spectrometry proteomics data have been deposited to the ProteomeXchange Consortium via the PRIDE [68] partner repository with the dataset identifier PXD053569. At the moment, only reviewers have access by logging in to the PRIDE website using the following account details: Username: reviewer_pxd053569@ebi.ac.uk; Password: tFL7cxQDcfjO.

## Supplementary Materials

The following supporting information can be downloaded at: www.mdpi.com//10.3390/biom14101239/s1, File S1. Boxplots representing the abundances of each of the proteins according to the day, zone and condition of the sample. Table S1. List of the 800 proteins of the dataset and their Mapman annotations. Table S2. Clusters mapman. Table S3. *p* values and adjusted *p* values for each of the tested effects and calculated from the differential analysis. File S2. Map corresponding to several KEGG pathways encompassing differentially expressed proteins. Table S4. Matrix representing pairs of co-expressed proteins in WW condition. Table S5. Matrix representing pairs of co-expressed proteins in WD condition. Table S6. Correspondance table between protein names, KO compounds and KEGG pathways. Table S7. List of high-degree nodes whose connectivities changed between WW and WD deficit condition.

## Abbreviations

The following abbreviations are used in this manuscript:

WD	water deficit
WW	well watered
PCA	principal component analysis
DEP	differentially expressed protein
SE	silk emergence

## References

[1] Erenstein O., Chamberlin J., Sonder K. (2021). Estimating the global number and distribution of maize and wheat farms. Glob. Food Secur..

[2] Erenstein O., Jaleta M., Sonder K., Mottaleb K., Prasanna B. (2022). Global maize production, consumption and trade: Trends and R&D implications. Food Secur..

[3] Vicente-Serrano S.M., Peña-Angulo D., Beguería S., Domínguez-Castro F., Tomás-Burguera M., Noguera I., Gimeno-Sotelo L., El Kenawy A. (2022). Global drought trends and future projections. Philos. Trans. R. Soc. A Math. Phys. Eng. Sci..

[4] Tesfaye K., Kruseman G., Cairns J.E., Zaman-Allah M., Wegary D., Zaidi P.H., Boote K.J., Rahut D., Erenstein O. (2018). Potential benefits of drought and heat tolerance for adapting maize to climate change in tropical environments. Clim. Risk Manag..

[5] Tang Y., Guo J., Jagadish S.V.K., Yang S., Qiao J., Wang Y., Xie K., Wang H., Yang Q., Deng L. (2023). Ovary abortion in field-grown maize under water-deficit conditions is determined by photo-assimilation supply. Field Crop. Res..

[6] Qi F., Zhang F. (2020). Cell Cycle Regulation in the Plant Response to Stress. Front. Plant Sci..

[7] Serna L. (2020). Maize stomatal responses against the climate change. Front. Plant Sci..

[8] Maazou A.R.S., Tu J., Qiu J., Liu Z. (2016). Breeding for Drought Tolerance in Maize (*Zea mays* L.). Am. J. Plant Sci..

[9] Claassen M.M., Shaw R.H. (1970). Water Deficit Effects on Corn. II. Grain Components. Agron. J..

[10] Westgate M.E., Grant D.L. (1989). Water deficits and reproduction in maize: Response of the reproductive tissue to water deficits at anthesis and mid-grain fill. Plant Physiol..

[11] Oury V., Tardieu F., Turc O. (2016). Ovary Apical Abortion under Water Deficit Is Caused by Changes in Sequential Development of Ovaries and in Silk Growth Rate in Maize. Plant Physiol..

[12] Schussler J.R., Westgate M.E. (1995). Assimilate Flux Determines Kernel Set at Low Water Potential in Maize. Maize Crop Sci..

[13] Turc O., Bouteillé M., Fuad-Hassan A., Welcker C., Tardieu F. (2016). The growth of vegetative and reproductive structures (leaves and silks) respond similarly to hydraulic cues in maize. New Phytol..

[14] Fuad-Hassan A., Tardieu F., Turc O. (2008). Drought-induced changes in anthesis-silking interval are related to silk expansion: A spatio-temporal growth analysis in maize plants subjected to soil water deficit. Plant Cell Environ..

[15] Sah R.P., Chakraborty M., Prasad K., Pandit M., Tudu V.K., Chakravarty M.K., Narayan S.C., Rana M., Moharana D. (2020). Impact of water deficit stress in maize: Phenology and yield components. Sci. Rep..

[16] Ribaut J.M., Hoisington D.A., Deutsch J.A., Jiang C., Gonzalez-de Leon D. (1996). Identification of quantitative trait loci under drought conditions in tropical maize. 1. Flowering parameters and the anthesis-silking interval. Theoret. Appl. Genet..

[17] Frova C., Krajewski P., di Fonzo N., Villa M., Sari-Gorla M. (1999). Genetic analysis of drought tolerance in maize by molecular markersI. Yield components. Theor. Appl. Genet..

[18] Semagn K., Beyene Y., Warburton M.L., Tarekegne A., Mugo S., Meisel B., Sehabiague P., Prasanna B.M. (2013). Meta-analyses of QTL for grain yield and anthesis silking interval in 18 maize populations evaluated under water-stressed and well-watered environments. BMC Genom..

[19] Almeida G.D., Makumbi D., Magorokosho C., Nair S., Borém A., Ribaut J.M., Bänziger M., Prasanna B.M., Crossa J., Babu R. (2012). QTL mapping in three tropical maize populations reveals a set of constitutive and adaptive genomic regions for drought tolerance. Theor. Appl. Genet..

[20] Evans M.M. (2007). The indeterminate gametophyte1 Gene of Maize Encodes a LOB Domain Protein Required for Embryo Sac and Leaf Development. Plant Cell.

[21] Sheridan W.F., Avalkina N.A., Shamrov I.I., Batyea T.B., Golubovskaya I.N. (1996). The *mac1* Gene: Controlling the Commitment to the Meiotic Pathway in Maize. Genetics.

[22] Drews G.N., Lee D., Christensen C.A. (1998). Genetic Analysis of Female Gametophyte Development and Function. Plant Cell.

[23] Pawlowski W.P., Wang C.J.R., Golubovskaya I.N., Szymaniak J.M., Shi L., Hamant O., Zhu T., Harper L., Sheridan W.F., Cande W.Z. (2009). Maize AMEIOTIC1 is essential for multiple early meiotic processes and likely required for the initiation of meiosis. Proc. Natl. Acad. Sci. USA.

[24] Mäkelä P., McLaughlin J.E., Boyer J.S. (2005). Imaging and Quantifying Carbohydrate Transport to the Developing Ovaries of Maize. Ann. Bot..

[25] Zinselmeier C., Habben J.E., Westgate M.E., Boyer J.S. (2000). Carbohydrate Metabolism in Setting and Aborting Maize Ovaries. Physiology and Modeling Kernel Set in Maize.

[26] McLaughlin J.E., Boyer J.S. (2004). Sugar-responsive Gene Expression, Invertase Activity, and Senescence in Aborting Maize Ovaries at Low Water Potentials. Ann. Bot..

[27] Zinselmeier C., Jeong B.R., Boyer J.S. (1999). Starch and the Control of Kernel Number in Maize at Low Water Potentials. Plant Physiol..

[28] Zinselmeier C., Westgate M.E., Schussler J.R., Jones R.J. (1995). Low Water Potential Disrupts Carbohydrate Metabolism in Maize (*Zea mays* L.) Ovaries. Plant Physiol..

[29] Boyle M.G., Boyer J.S., Morgan P.W. (1991). Stem Infusion of Liquid Culture Medium Prevents Reproductive Failure of Maize at Low Water Potential. Crop. Sci..

[30] Zhou J., Tian L., Wang S., Li H., Zhao Y., Zhang M., Wang X., An P., Li C. (2021). Ovary Abortion Induced by Combined Waterlogging and Shading Stress at the Flowering Stage Involves Amino Acids and Flavonoid Metabolism in Maize. Front. Plant Sci..

[31] McLaughlin J.E., Boyer J.S. (2004). Glucose Localization in Maize Ovaries When Kernel Number Decreases at Low Water Potential and Sucrose is Fed to the Stems. Ann. Bot..

[32] Wang Y., Wang J., Guo H., Wu X., Hao M., Zhang R. (2023). Integrative transcriptome and metabolome analysis reveals the mechanism of exogenous melatonin alleviating drought stress in maize roots. Plant Physiol. Biochem..

[33] Sun J., Wang Y., Zhang X., Cheng Z., Song Y., Li H., Wang N., Liu S., Cao Z., Li H. (2024). Transcriptomic and Metabolomic Analyses Reveal the Role of Phenylalanine Metabolism in the Maize Response to Stalk Rot Caused by *Fusarium proliferatum*. Int. J. Mol. Sci..

[34] Schnable P.S., Ware D., Fulton R.S., Stein J.C., Wei F., Pasternak S., Liang C., Zhang J., Fulton L., Graves T.A. (2009). The B73 maize genome: Complexity, diversity, and dynamics. Science.

[35] Balliau T., Blein-Nicolas M., Zivy M. (2018). Evaluation of Optimized Tube-Gel Methods of Sample Preparation for Large-Scale Plant Proteomics. Proteomes.

[36] Blein-Nicolas M., Albertin W., Valot B., Marullo P., Sicard D., Giraud C., Huet S., Bourgais A., Dillmann C., de Vienne D. (2013). Yeast Proteome Variations Reveal Different Adaptive Responses to Grape Must Fermentation. Mol. Biol. Evol..

[37] Craig R., Beavis R.C. (2004). TANDEM: Matching proteins with tandem mass spectra. Bioinformatics.

[38] Langella O., Valot B., Balliau T., Blein-Nicolas M., Bonhomme L., Zivy M. (2016). X!TandemPipeline: A Tool to Manage Sequence Redundancy for Protein Inference and Phosphosite Identification. J. Proteome Res..

[39] Valot B., Langella O., Nano E., Zivy M. (2011). MassChroQ: A versatile tool for mass spectrometry quantification. Proteomics.

[40] Millan-Oropeza A., Henry C., Blein-Nicolas M., Aubert-Frambourg A., Moussa F., Bleton J., Virolle M.J. (2017). Quantitative Proteomics Analysis Confirmed Oxidative Metabolism Predominates in Streptomyces coelicolor versus Glycolytic Metabolism in Streptomyces lividans. J. Proteome Res..

[41] R Foundation for Statistical Computing (2022). R: A Language and Environment for Statistical Computing. https://www.R-project.org.

[42] Bolger M., Schwacke R., Usadel B. (2021). MapMan Visualization of RNA-Seq Data Using Mercator4 Functional Annotations. Methods Mol. Biol..

[43] Benjamini Y., Hochberg Y. (1995). Controlling the False Discovery Rate: A Practical and Powerful Approach to Multiple Testing. J. R. Stat. Soc. Ser. B Methodol..

[44] Chen Y., Du T., Zhang J., Chen S., Fu J., Li H., Yang Q. (2023). Genes and pathways correlated with heat stress responses and heat tolerance in maize kernels. Plant Sci..

[45] Zi X., Zhou S., Wu B. (2022). Alpha-Linolenic Acid Mediates Diverse Drought Responses in Maize (*Zea mays* L.) at Seedling and Flowering Stages. Molecules.

[46] Gao X., Fu X., Song J., Zhang Y., Cui X., Su C., Ge L., Shao J., Xin L., Saarikettu J. (2015). Poly(A)+ mRNA-binding protein Tudor-SN regulates stress granules aggregation dynamics. FEBS J..

[47] Gutierrez-Beltran E., Elander P.H., Dalman K., Dayhoff G.W., Moschou P.N., Uversky V.N., Crespo J.L., Bozhkov P.V. (2021). Tudor staphylococcal nuclease is a docking platform for stress granule components and is essential for SnRK1 activation in Arabidopsis. EMBO J..

[48] Marcos R., Izquierdo Y., Vellosillo T., Kulasekaran S., Cascón T., Hamberg M., Castresana C. (2015). 9-Lipoxygenase-Derived Oxylipins Activate Brassinosteroid Signaling to Promote Cell Wall-Based Defense and Limit Pathogen Infection. Plant Physiol..

[49] Wang C., Xiang Y., Qian D. (2021). Current progress in plant V-ATPase: From biochemical properties to physiological functions. J. Plant Physiol..

[50] Osnato M. (2021). Expansin helps maize to keep the right timing: Inducible expression of an Expansin gene mitigates drought effects on grain yields. Plant Cell.

[51] Chen J., Xu W., Velten J., Xin Z., Stout J. (2012). Characterization of maize inbred lines for drought and heat tolerance. J. Soil Water Conserv..

[52] Shirinpour M., Atazadeh E., Bybordi A., Monirifar H., Amini A., Hossain M.A., Aharizad S., Asghari A. (2023). Gene action and inheritance of grain yield and root morphological traits in hybrid maize grown under water deficit conditions. S. Afr. J. Bot..

[53] Li P., Ma H., Xiao N., Zhang Y., Xu T., Xia T. (2023). Overexpression of the ZmSUS1 gene alters the content and composition of endosperm starch in maize (*Zea mays* L.). Planta.

[54] Bargmann B.O., Munnik T. (2006). The role of phospholipase D in plant stress responses. Curr. Opin. Plant Biol..

[55] Niu L., Wang W., Li Y., Wu X., Wang W. (2024). Maize multi-omics reveal leaf water status controlling of differential transcriptomes, proteomes and hormones as mechanisms of age-dependent osmotic stress response in leaves. Stress Biol..

[56] Jagtap A.B., Yadav I.S., Vikal Y., Praba U.P., Kaur N., Gill A.S., Johal G.S. (2023). Transcriptional dynamics of maize leaves, pollens and ovules to gain insights into heat stress-related responses. Front. Plant Sci..

[57] Danilevskaya O.N., Yu G., Meng X., Xu J., Stephenson E., Estrada S., Chilakamarri S., Zastrow-Hayes G., Thatcher S. (2019). Developmental and transcriptional responses of maize to drought stress under field conditions. Plant Direct.

[58] Li X., Wu J., Yi F., Lai J., Chen J. (2022). High temporal-resolution transcriptome landscapes of maize embryo sac and ovule during early seed development. Plant Mol. Biol..

[59] Navarrete F., Gallei M., Kornienko A.E., Saado I., Khan M., Chia K.S., Darino M.A., Bindics J., Djamei A. (2021). TOPLESS promotes plant immunity by repressing auxin signaling and is targeted by the fungal effector Naked1. Plant Commun..

[60] Liu L., Yahaya B.S., Li J., Wu F. (2024). Enigmatic role of auxin response factors in plant growth and stress tolerance. Front. Plant Sci..

[61] Nemhauser J.L., Mockler T.C., Chory J. (2004). Interdependency of Brassinosteroid and Auxin Signaling in Arabidopsis. PLoS Biol..

[62] Kuczyńska A., Michałek M., Ogrodowicz P., Kempa M., Witaszak N., Dziurka M., Gruszka D., Daszkowska-Golec A., Szarejko I., Krajewski P. (2024). Drought-induced molecular changes in crown of various barley phytohormone mutants. Plant Signal. Behav..

[63] Wang Z., Mambelli S., Setter T.L. (2002). Abscisic acid catabolism in maize kernels in response to water deficit at early endosperm development. Ann. Bot..

[64] Setter T.L., Flannigan B.A. (2001). Water deficit inhibits cell division and expression of transcripts involved in cell proliferation and endoreduplication in maize endosperm. J. Exp. Bot..

[65] Andersen M.N., Asch F., Wu Y., Jensen C.R., Næsted H., Mogensen V.O., Koch K.E. (2002). Soluble Invertase Expression Is an Early Target of Drought Stress during the Critical, Abortion-Sensitive Phase of Young Ovary Development in Maize. Plant Physiol..

[66] Best N.B., Hartwig T., Budka J., Fujioka S., Johal G., Schulz B., Dilkes B.P. (2016). nana plant2 Encodes a Maize Ortholog of the Arabidopsis Brassinosteroid Biosynthesis Gene DWARF1, Identifying Developmental Interactions between Brassinosteroids and Gibberellins. Plant Physiol..

[67] Oh E., Zhu J.Y., Ryu H., Hwang I., Wang Z.Y. (2014). TOPLESS mediates brassinosteroid-induced transcriptional repression through interaction with BZR1. Nat. Commun..

[68] Perez-Riverol Y., Bai J., Bandla C., García-Seisdedos D., Hewapathirana S., Kamatchinathan S., Kundu D.J., Prakash A., Frericks-Zipper A., Eisenacher M. (2021). The PRIDE database resources in 2022: A hub for mass spectrometry-based proteomics evidences. Nucleic Acids Res..

