# Supplementary figures and images for: A Moderate Water Deficit Induces Profound Changes in the Proteome of Developing Maize Ovaries
Source: Biomolecules. 2024 Sep 30;14(10):1239. doi: 10.3390/biom14101239 (PMC11506675; doi:10.3390/biom14101239)

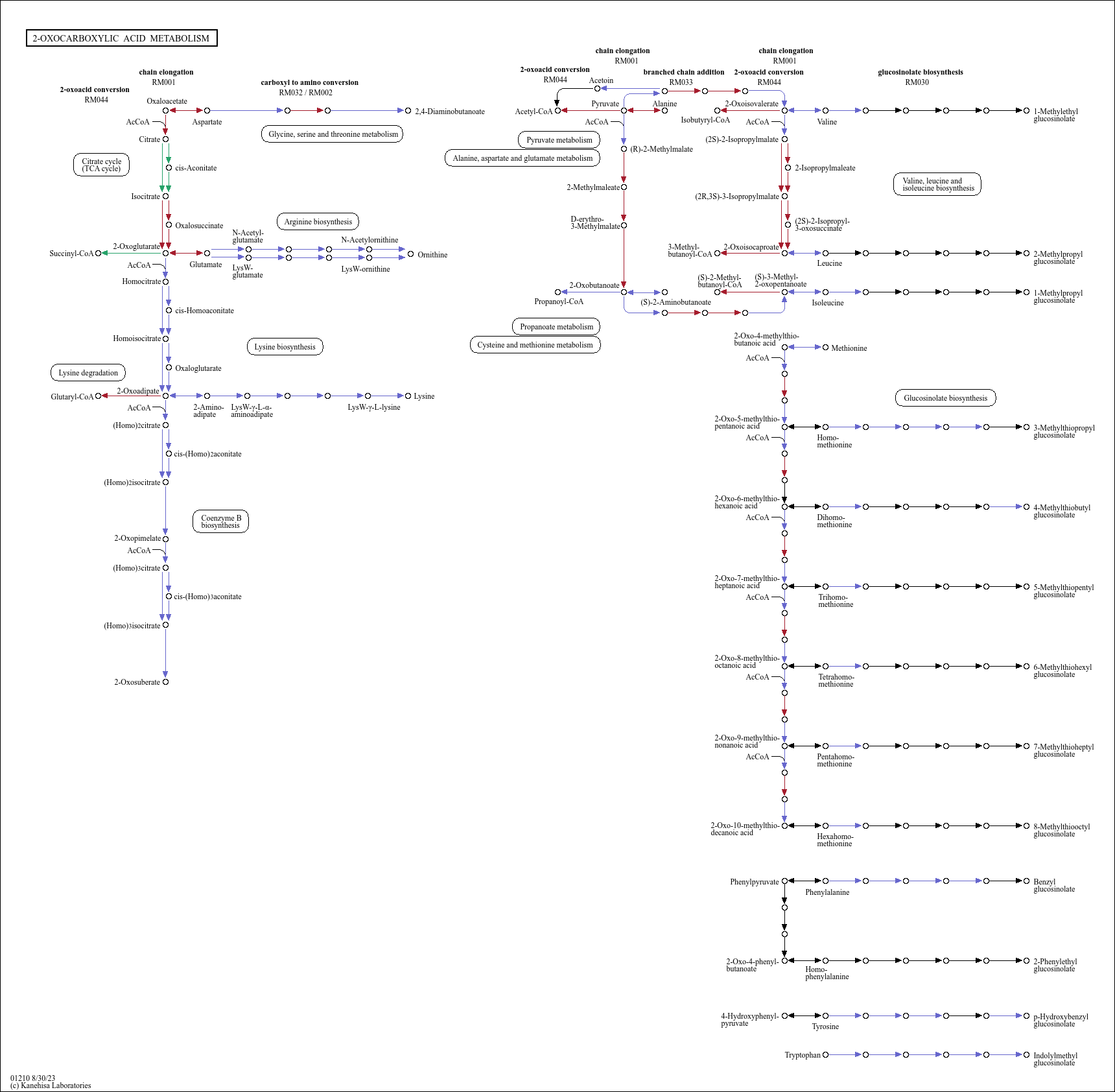

Supplement: Supplementary file 1 [file biomolecules-14-01239-s001.zip › File S2. KEGGpathways/2oxocarboxylic_acid_condition.png]

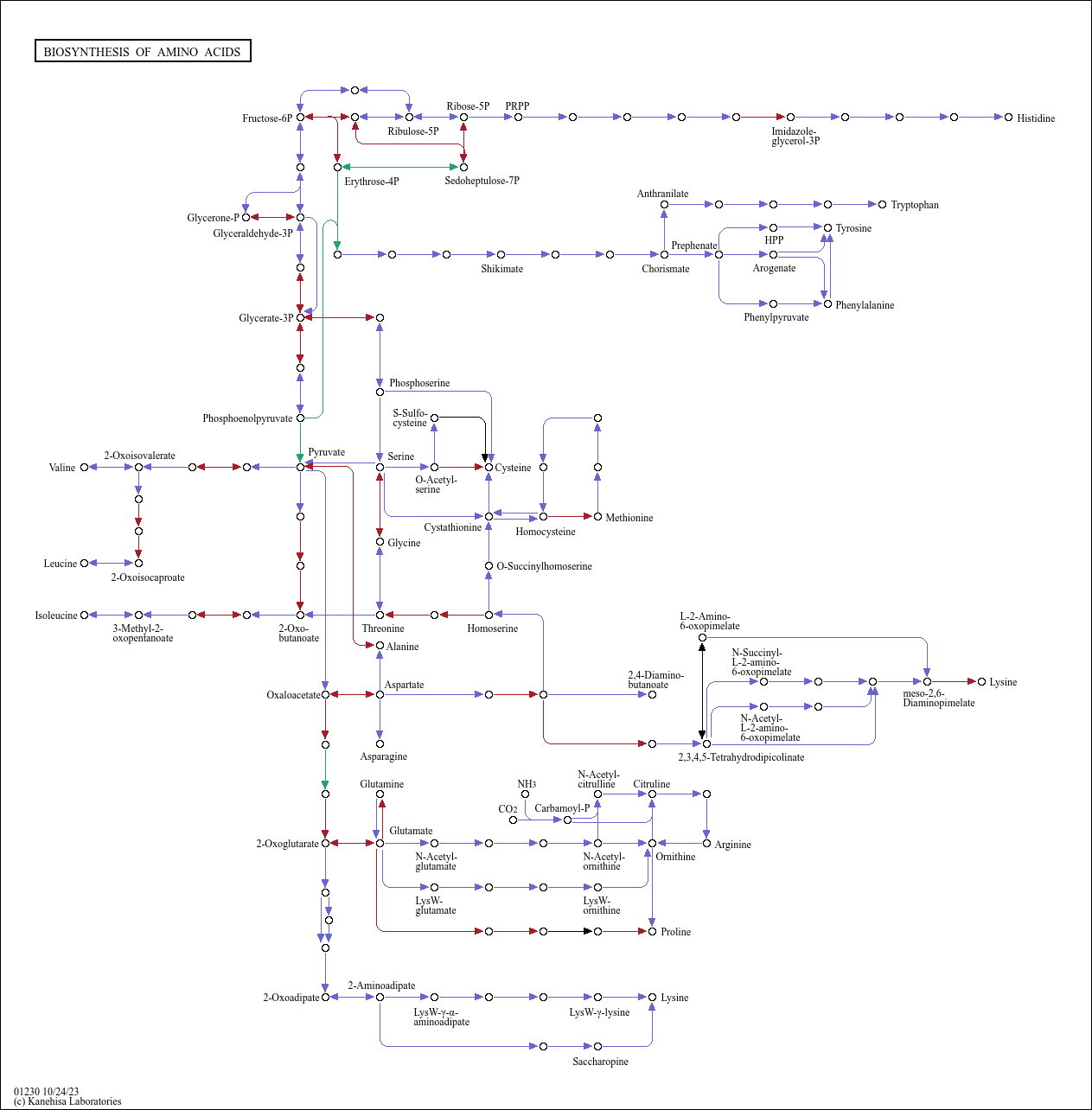

Supplement: Supplementary file 1 [file biomolecules-14-01239-s001.zip › File S2. KEGGpathways/aa_biosynthesis_condition.png]

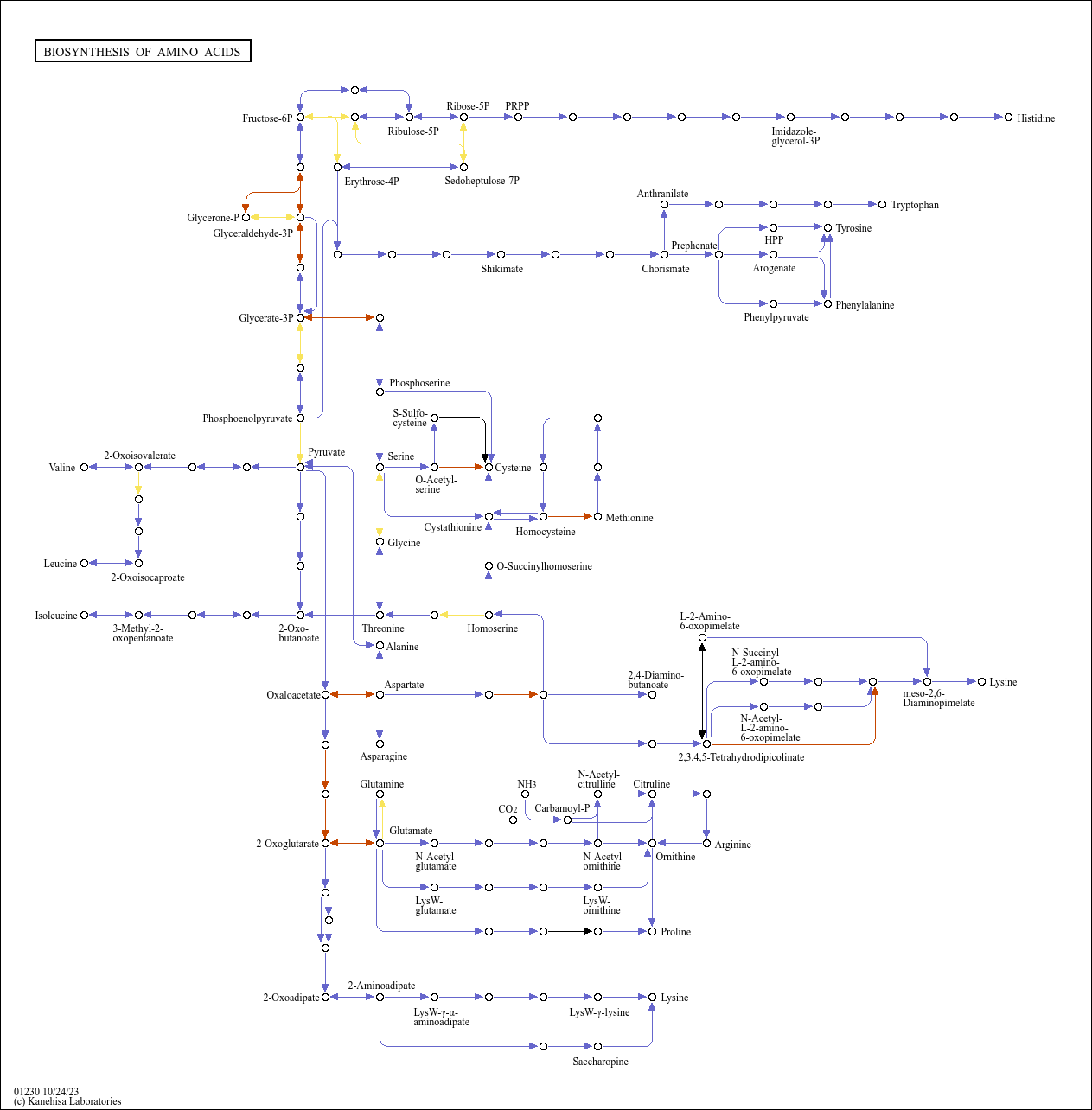

Supplement: Supplementary file 1 [file biomolecules-14-01239-s001.zip › File S2. KEGGpathways/aa_biosynthesis_day.png]

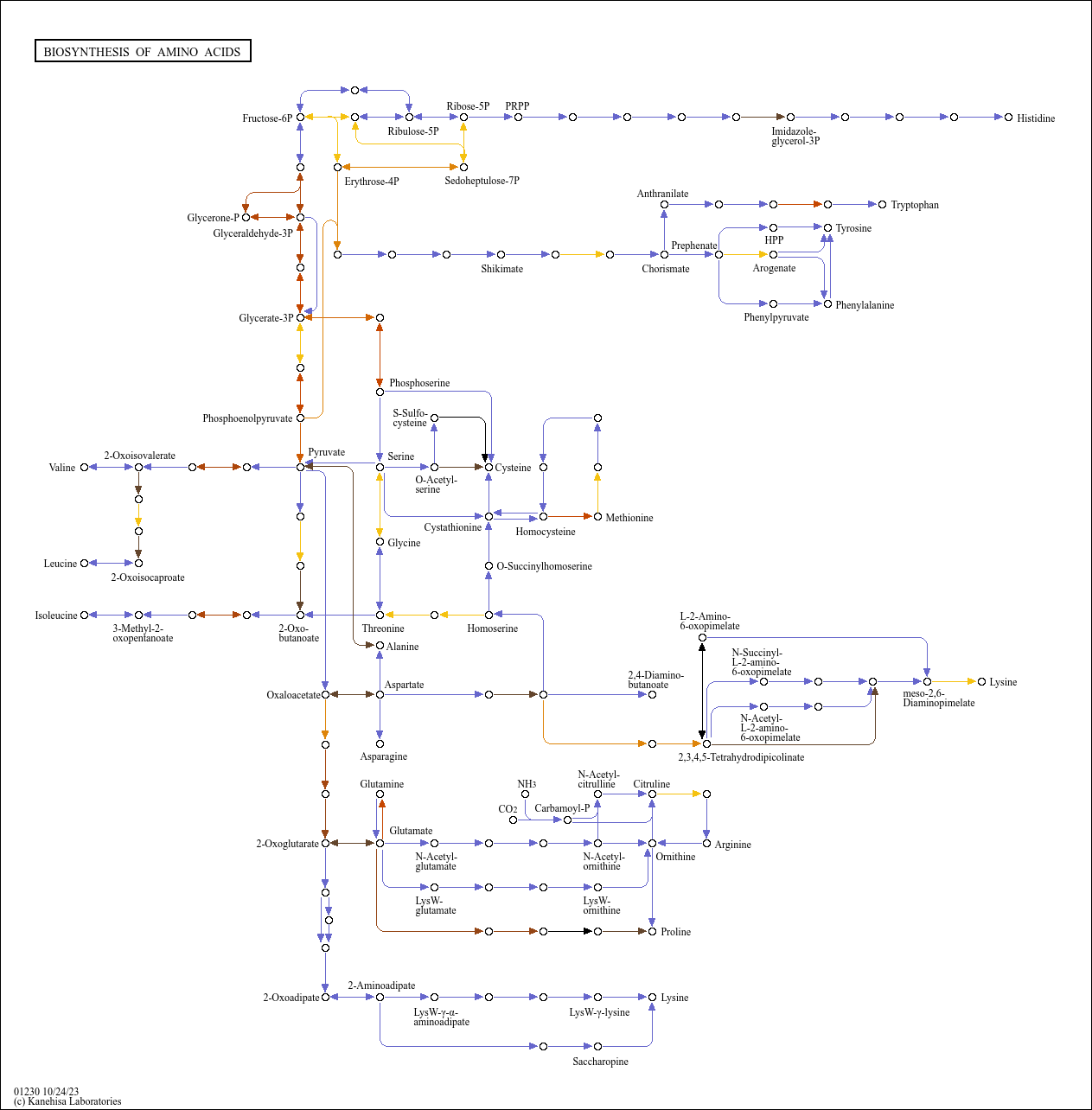

Supplement: Supplementary file 1 [file biomolecules-14-01239-s001.zip › File S2. KEGGpathways/aa_biosynthesis_zone.png]

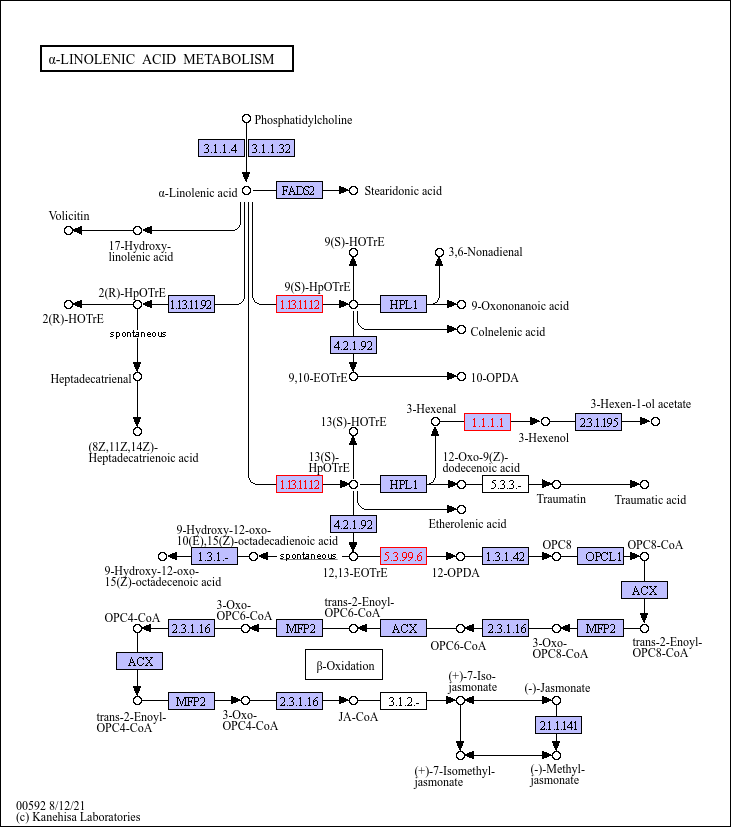

Supplement: Supplementary file 1 [file biomolecules-14-01239-s001.zip › File S2. KEGGpathways/alpha_linolenic_acide_dzI.png]

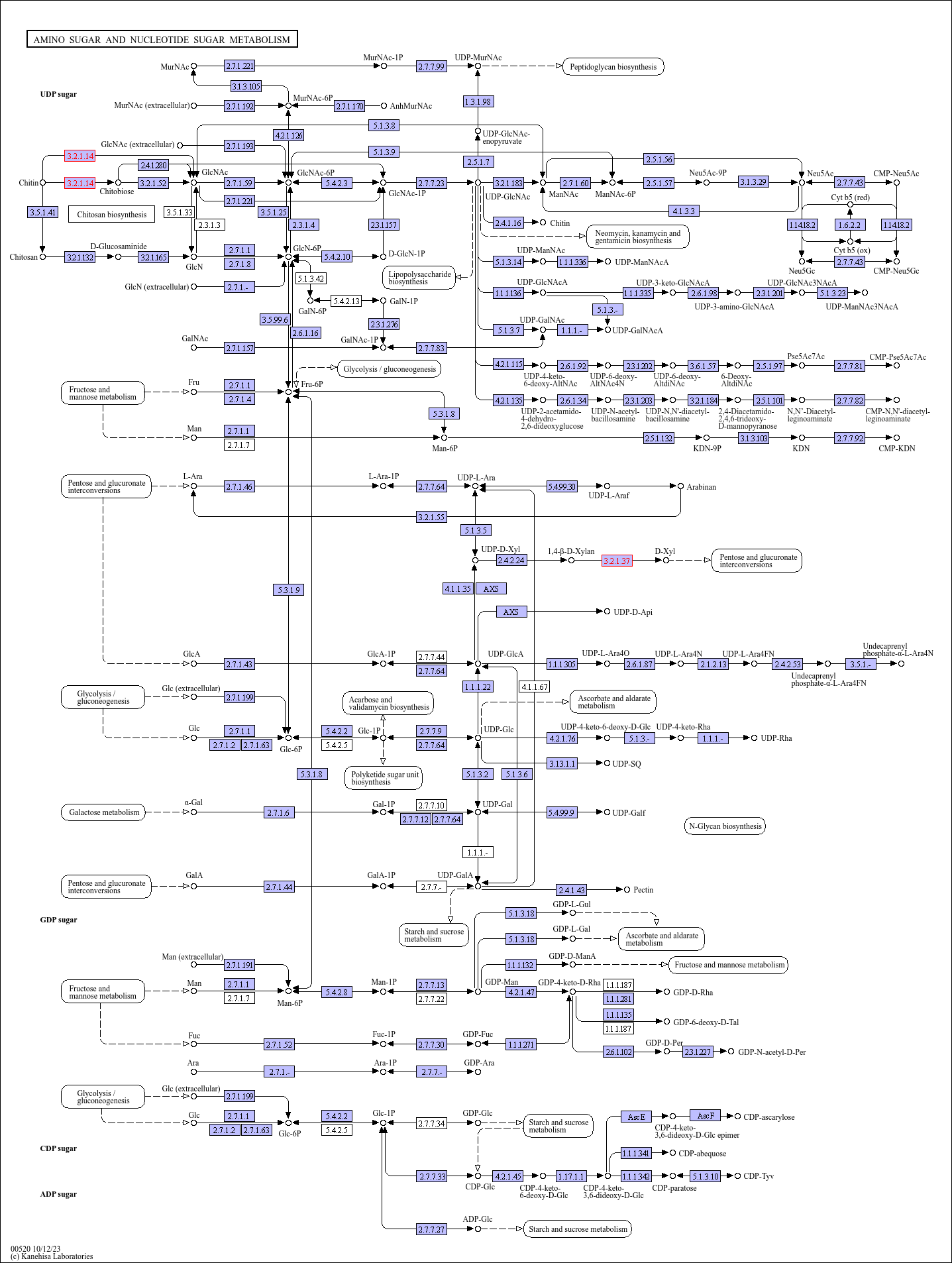

Supplement: Supplementary file 1 [file biomolecules-14-01239-s001.zip › File S2. KEGGpathways/amino_sugar_nt_sugar_metabo_dzI.png]

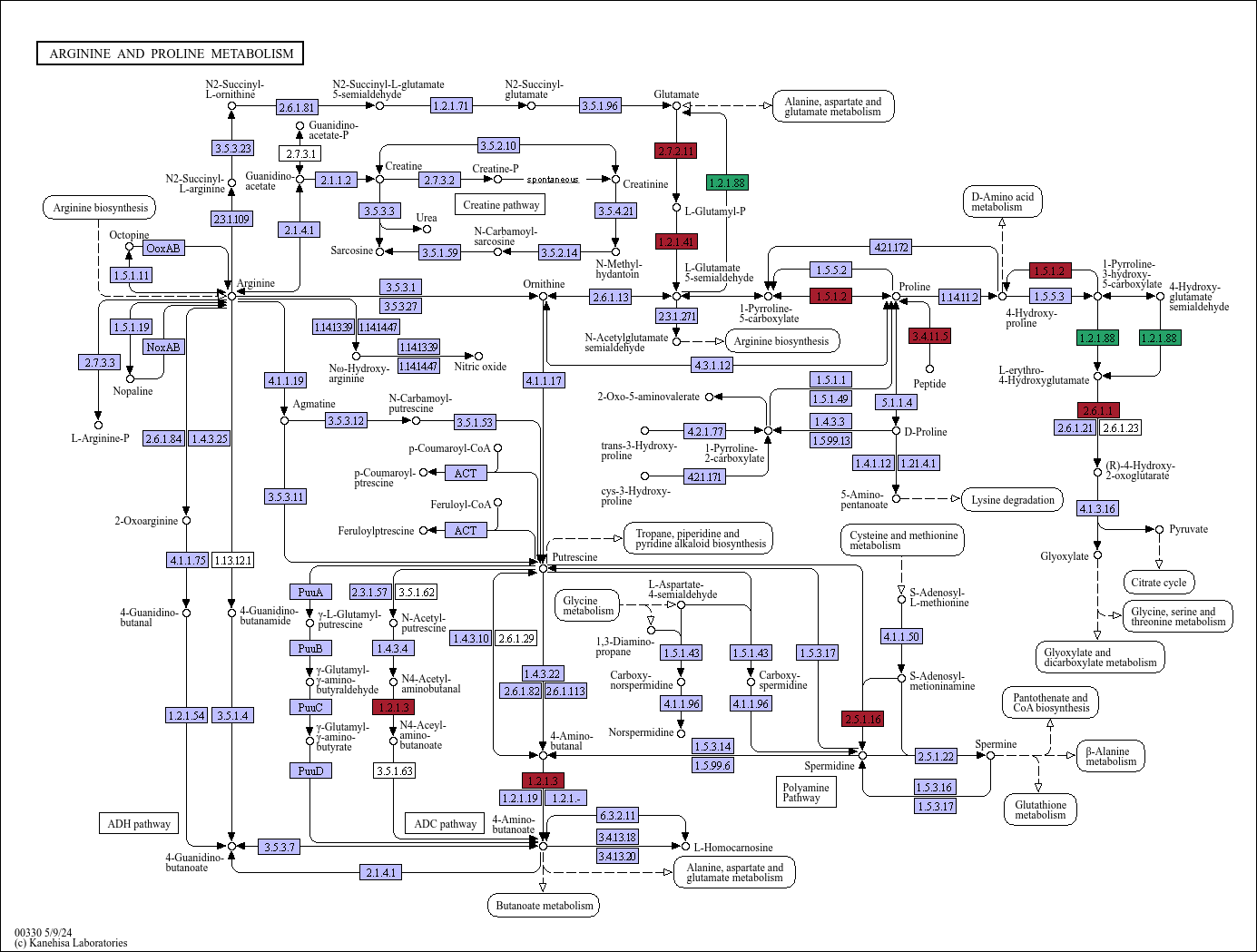

Supplement: Supplementary file 1 [file biomolecules-14-01239-s001.zip › File S2. KEGGpathways/arginine_proline_condition.png]

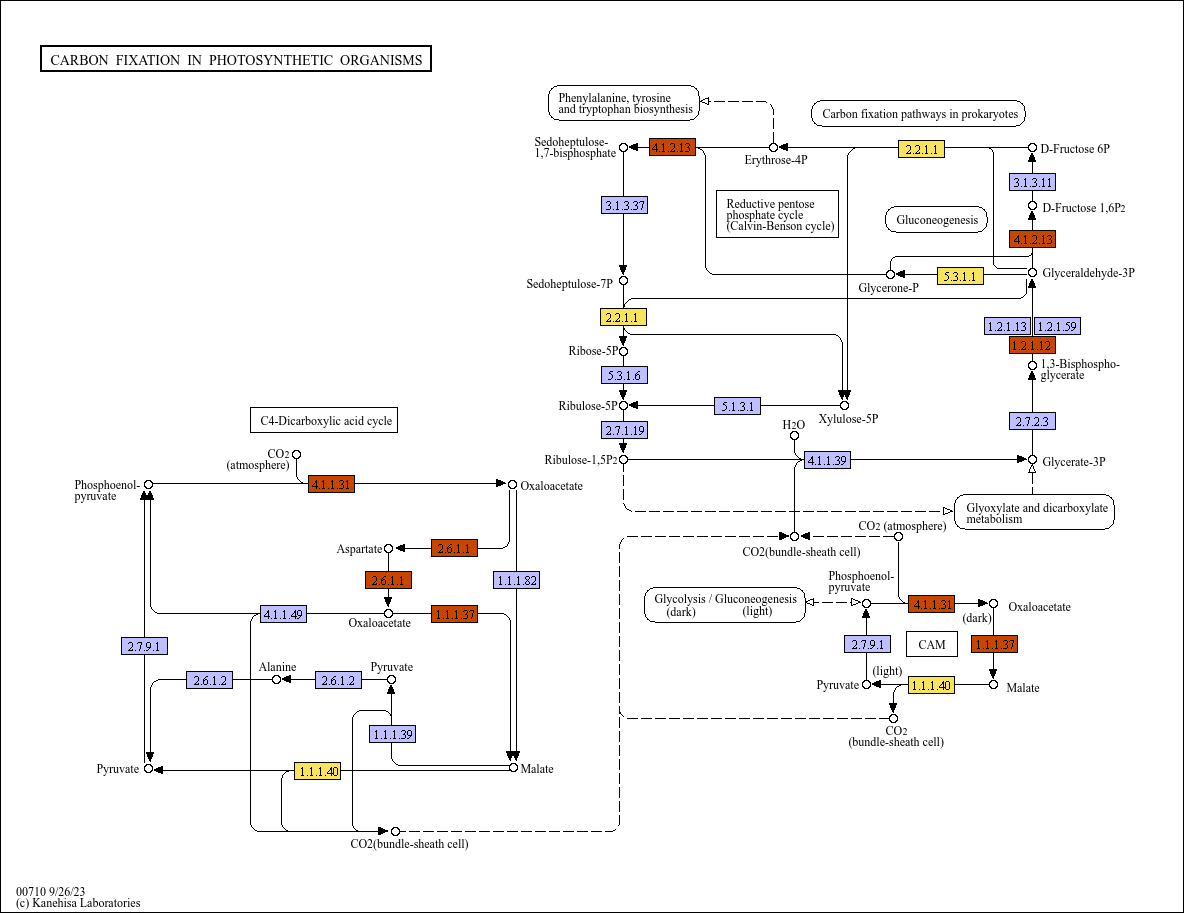

Supplement: Supplementary file 1 [file biomolecules-14-01239-s001.zip › File S2. KEGGpathways/carbon_fixation_day.png]

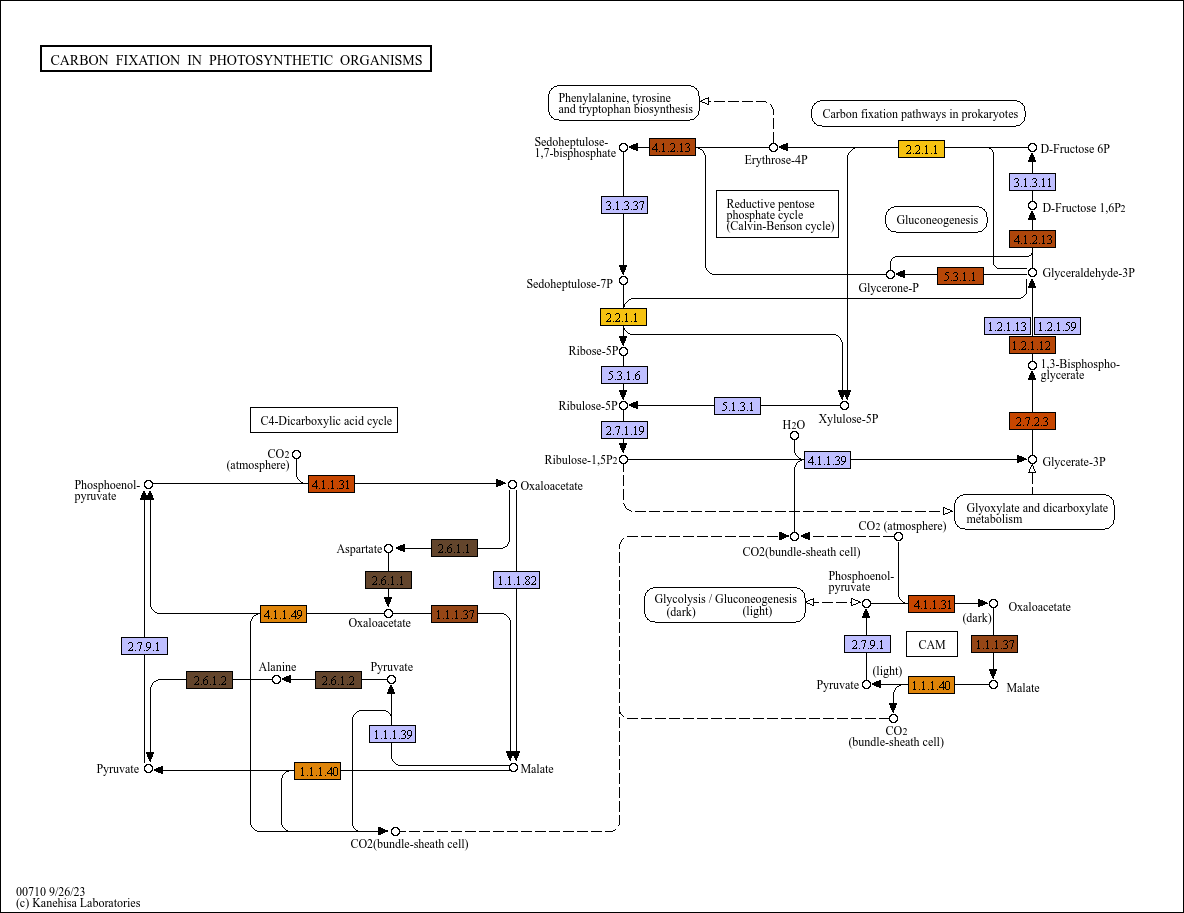

Supplement: Supplementary file 1 [file biomolecules-14-01239-s001.zip › File S2. KEGGpathways/carbon_fixation_zone.png]

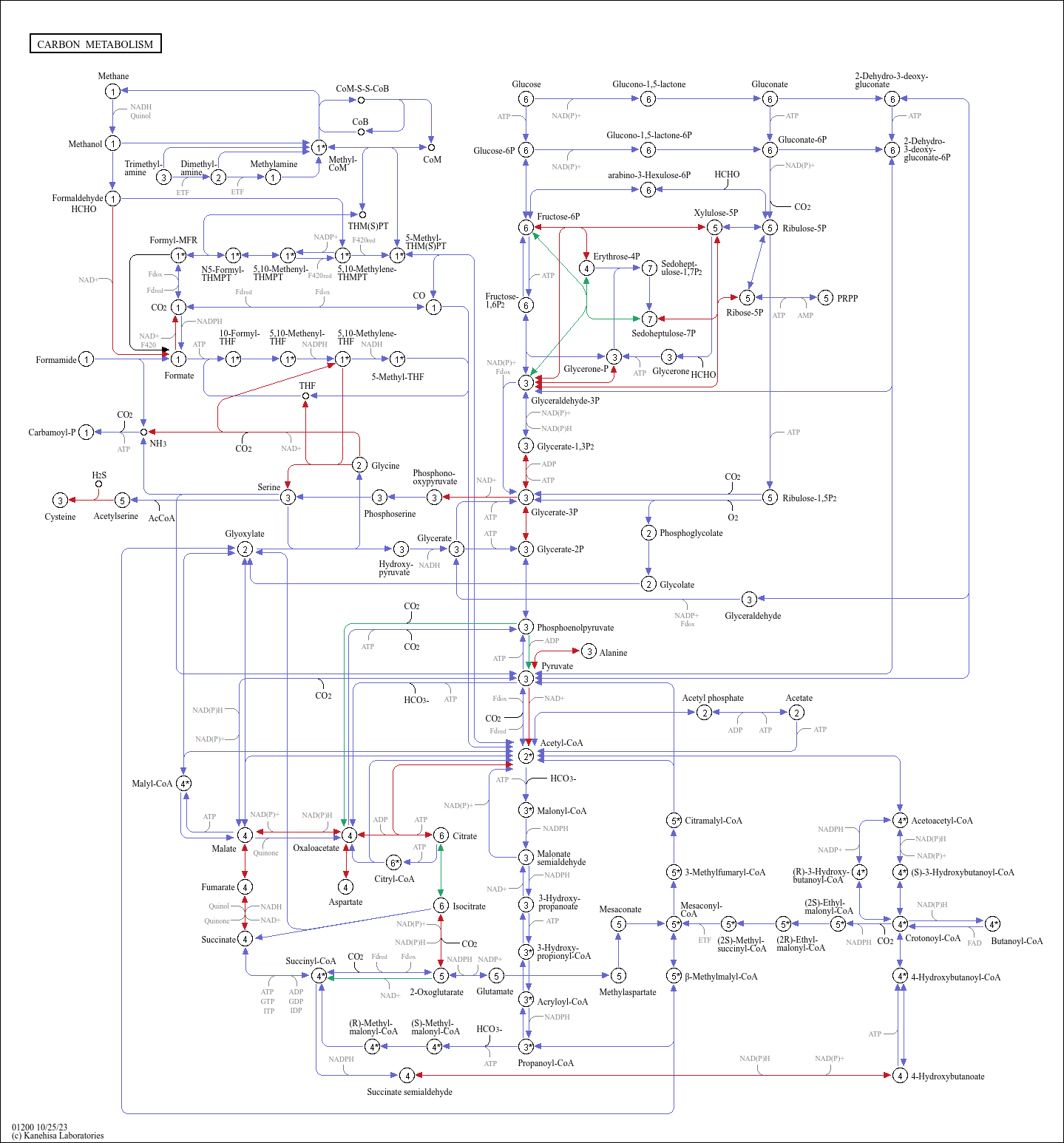

Supplement: Supplementary file 1 [file biomolecules-14-01239-s001.zip › File S2. KEGGpathways/carbon_metabo_condition.png]

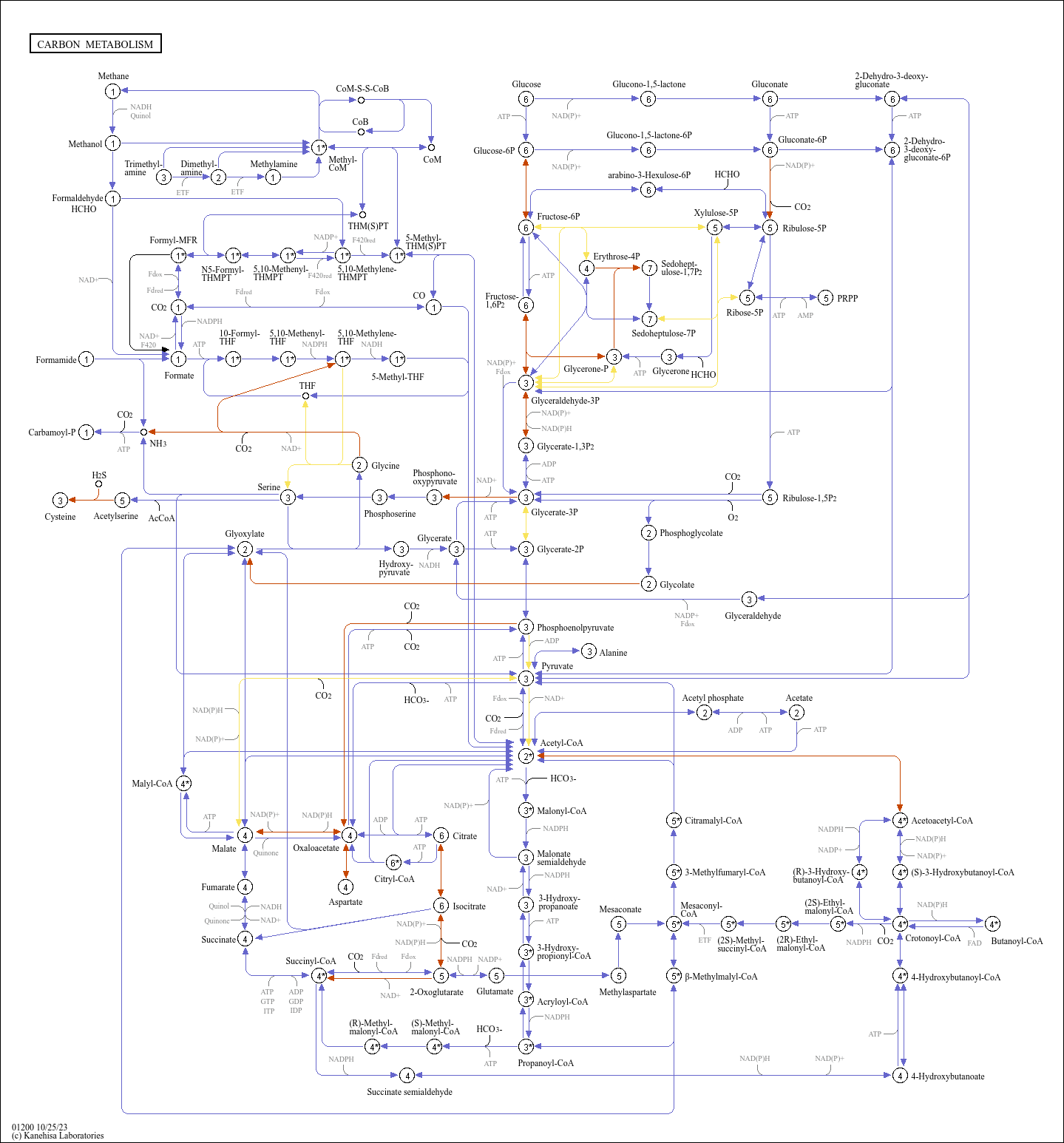

Supplement: Supplementary file 1 [file biomolecules-14-01239-s001.zip › File S2. KEGGpathways/carbon_metabo_day.png]

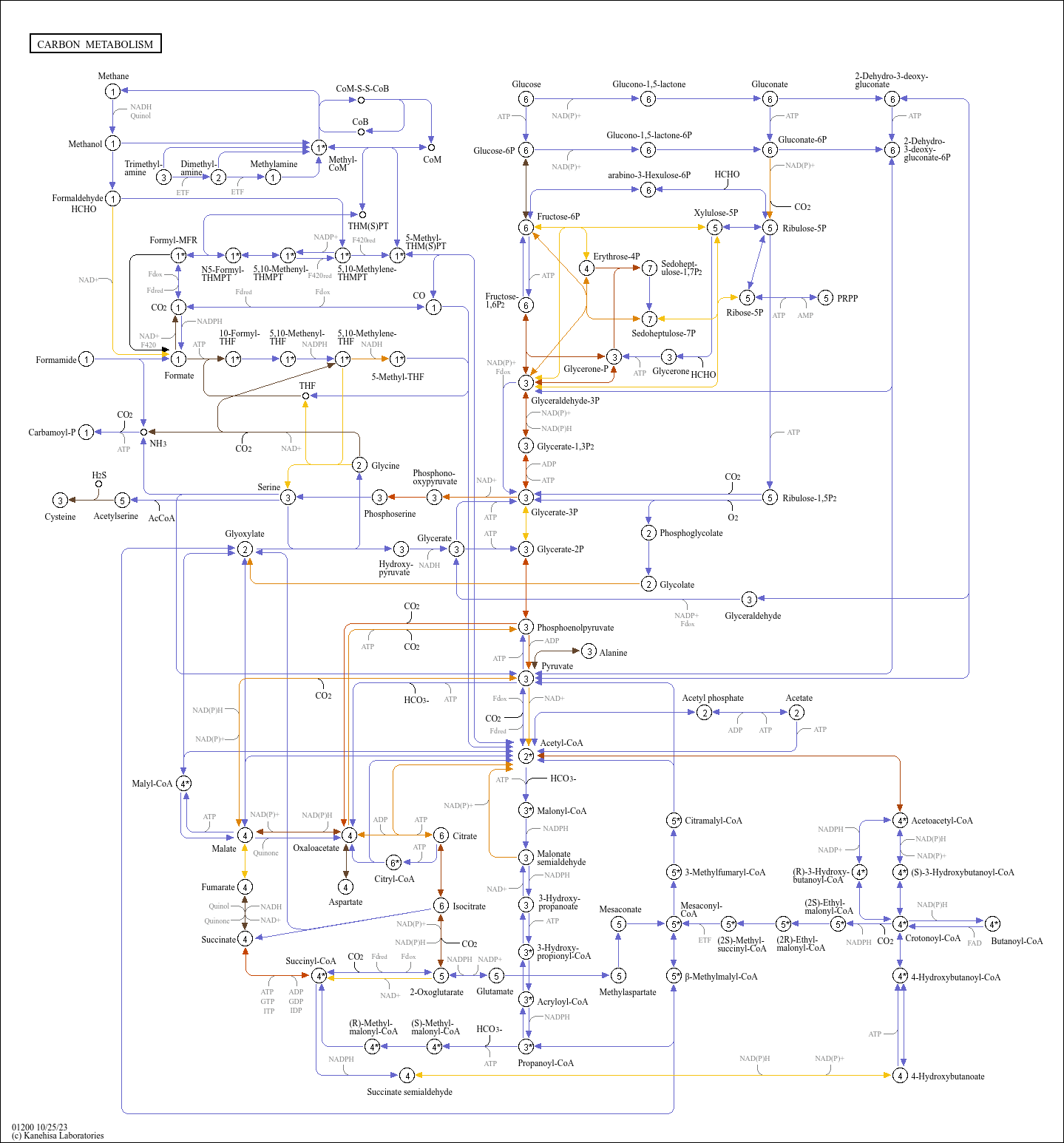

Supplement: Supplementary file 1 [file biomolecules-14-01239-s001.zip › File S2. KEGGpathways/carbon_metabo_zone.png]

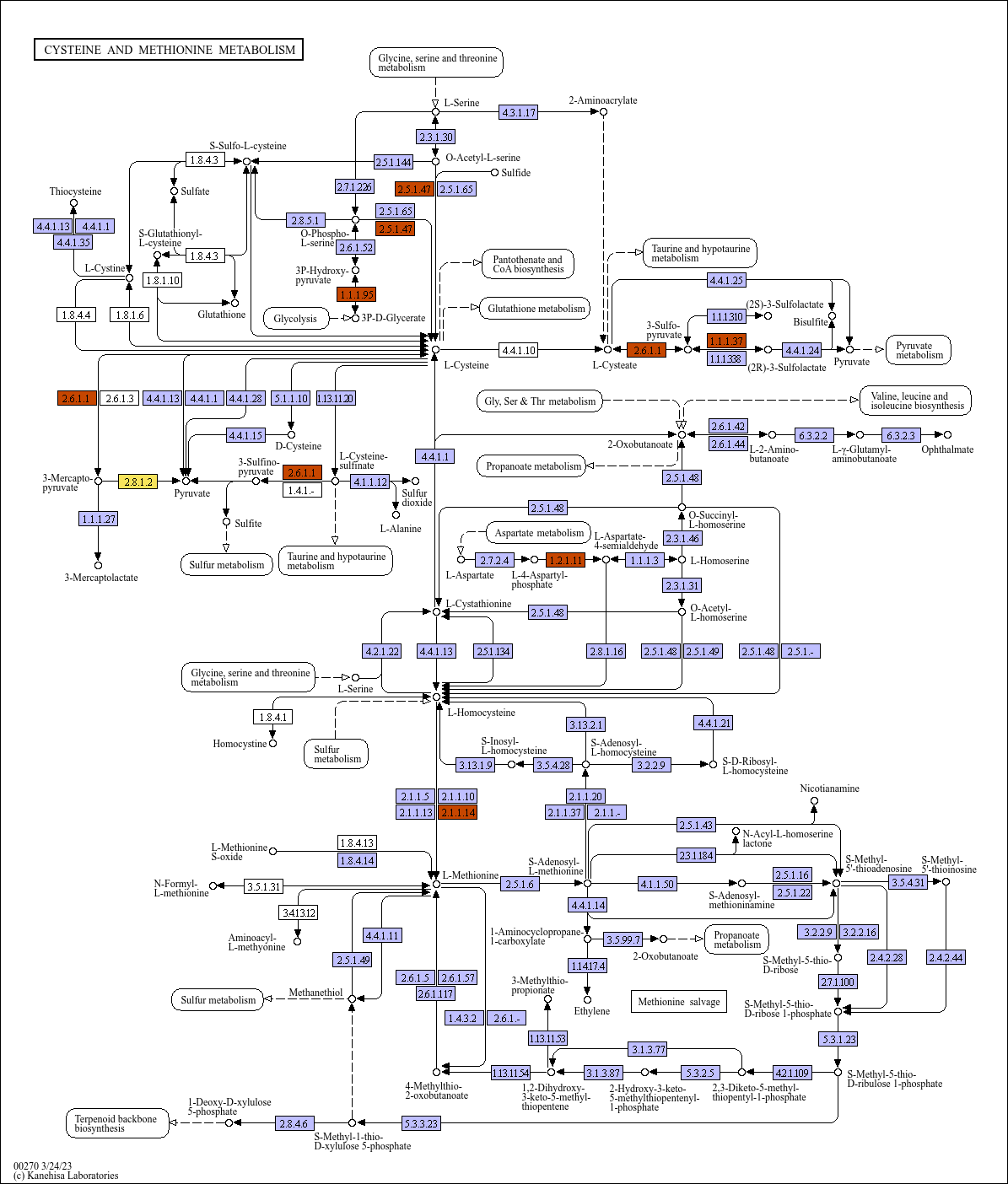

Supplement: Supplementary file 1 [file biomolecules-14-01239-s001.zip › File S2. KEGGpathways/cysteine_methionine_day.png]

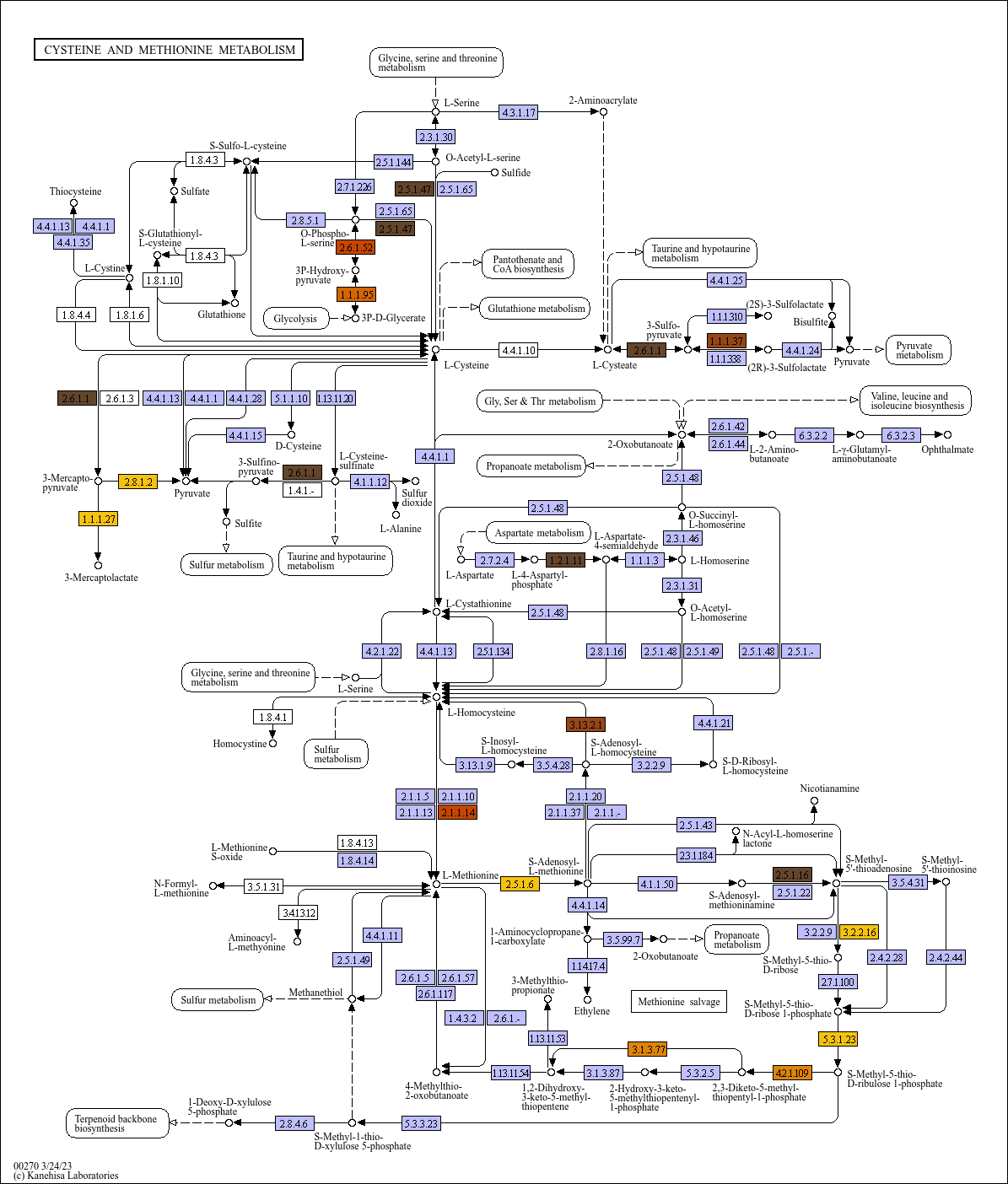

Supplement: Supplementary file 1 [file biomolecules-14-01239-s001.zip › File S2. KEGGpathways/cysteine_methionine_zone.png]

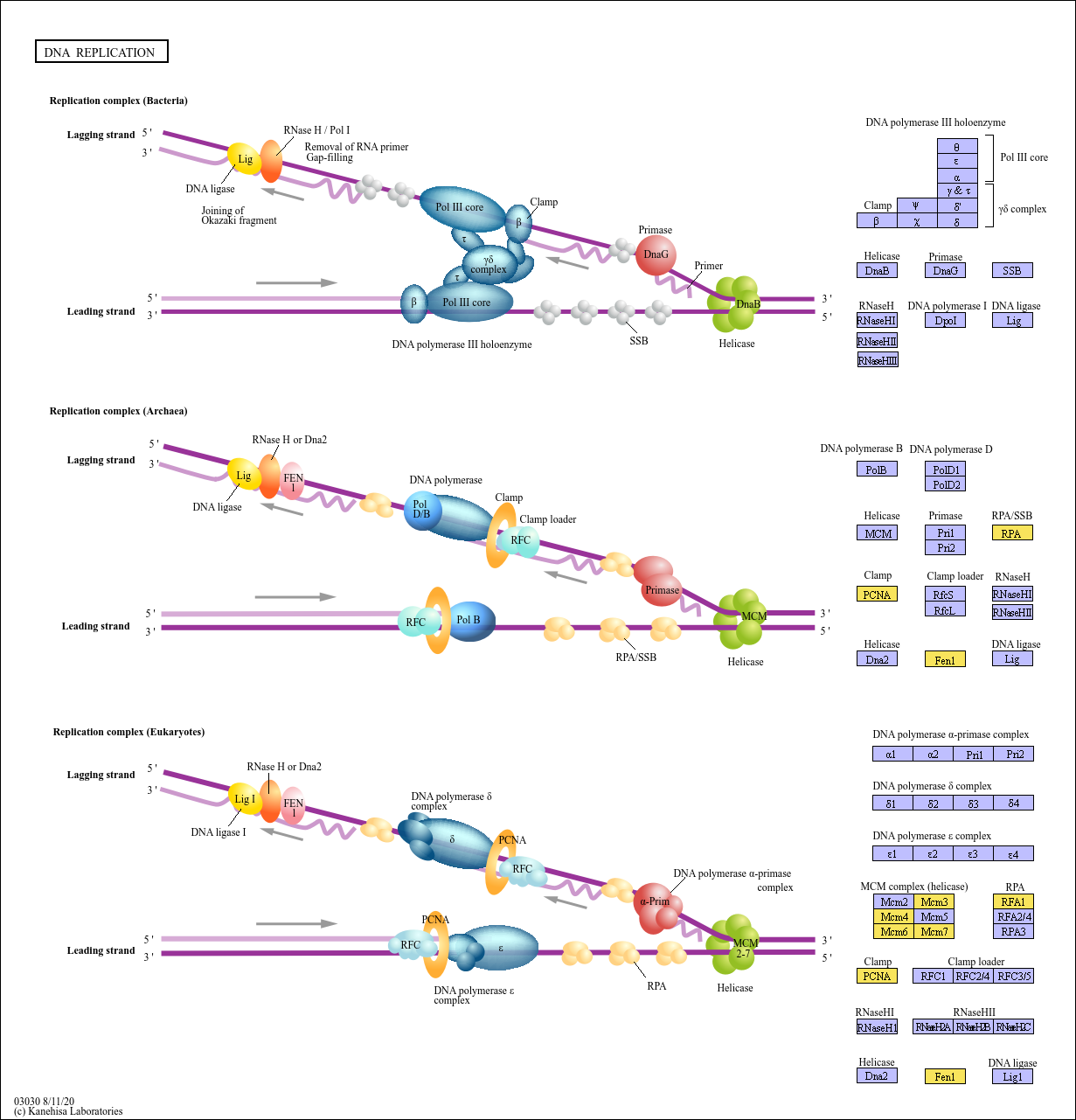

Supplement: Supplementary file 1 [file biomolecules-14-01239-s001.zip › File S2. KEGGpathways/DNA_replication_day.png]

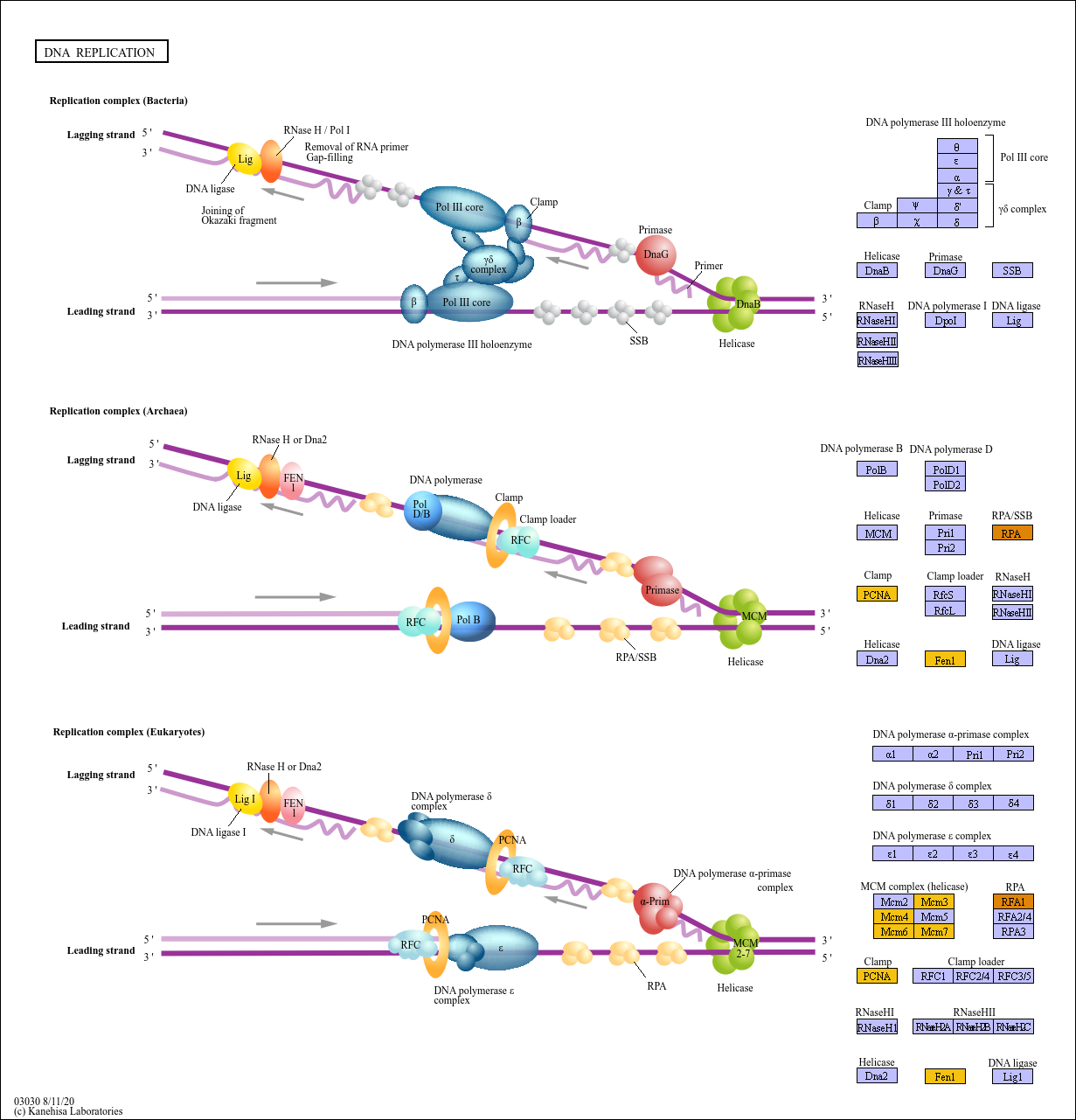

Supplement: Supplementary file 1 [file biomolecules-14-01239-s001.zip › File S2. KEGGpathways/DNA_replication_zone.png]

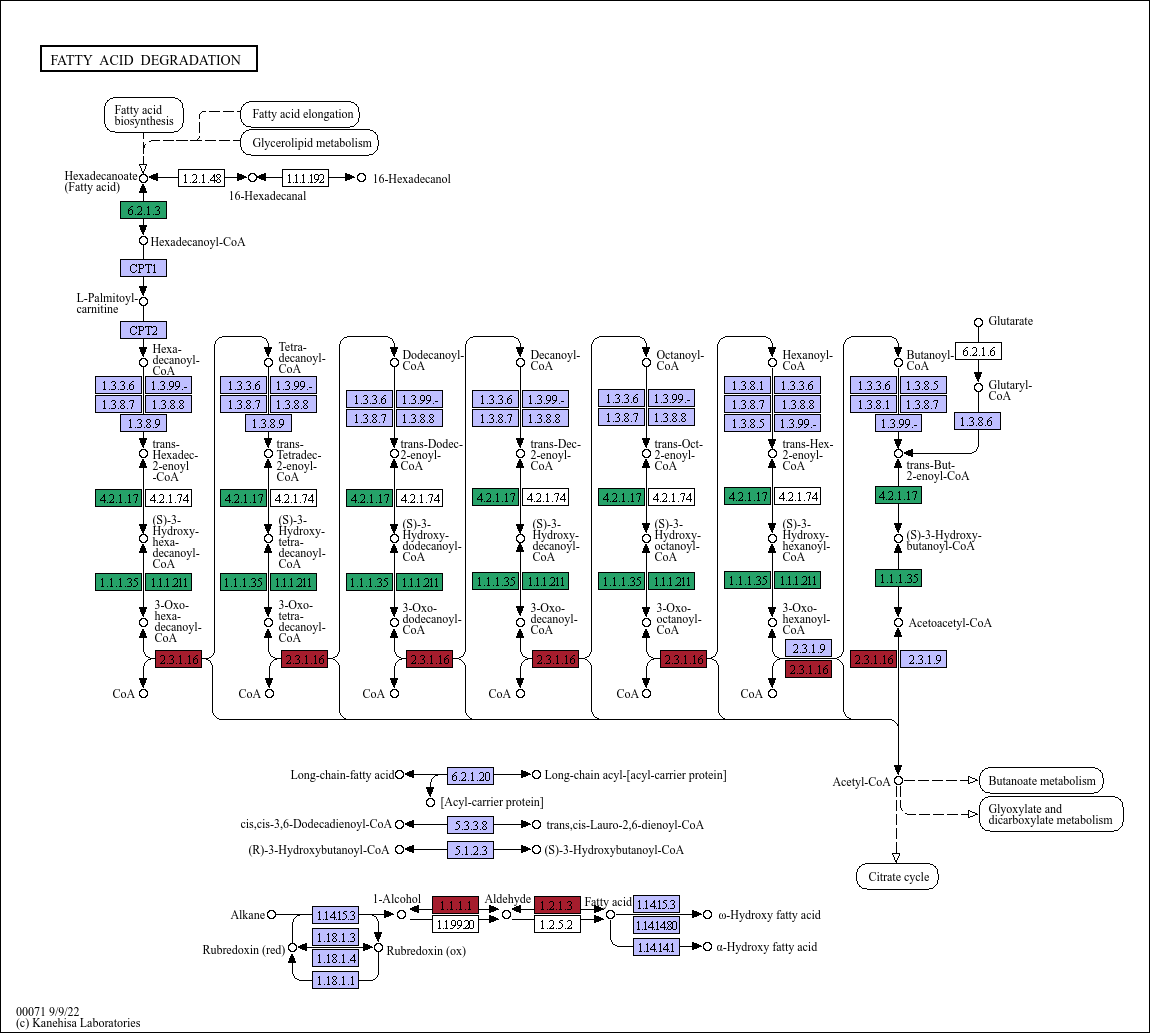

Supplement: Supplementary file 1 [file biomolecules-14-01239-s001.zip › File S2. KEGGpathways/fattyacid_degration_condition.png]

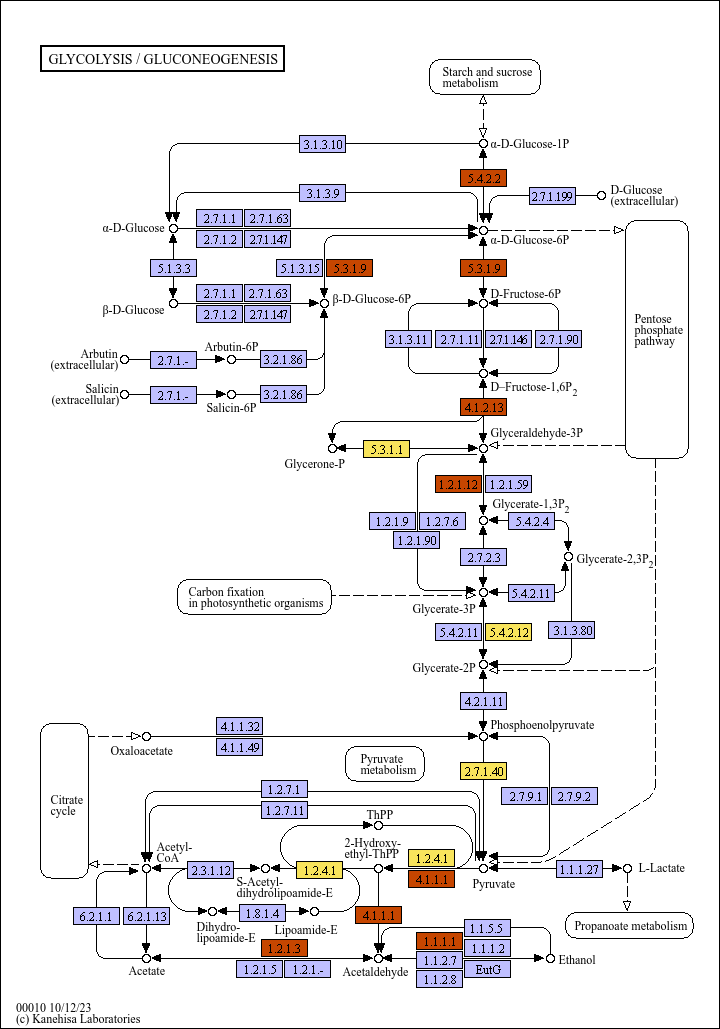

Supplement: Supplementary file 1 [file biomolecules-14-01239-s001.zip › File S2. KEGGpathways/glycolyse_gluconeogenesis_day.png]

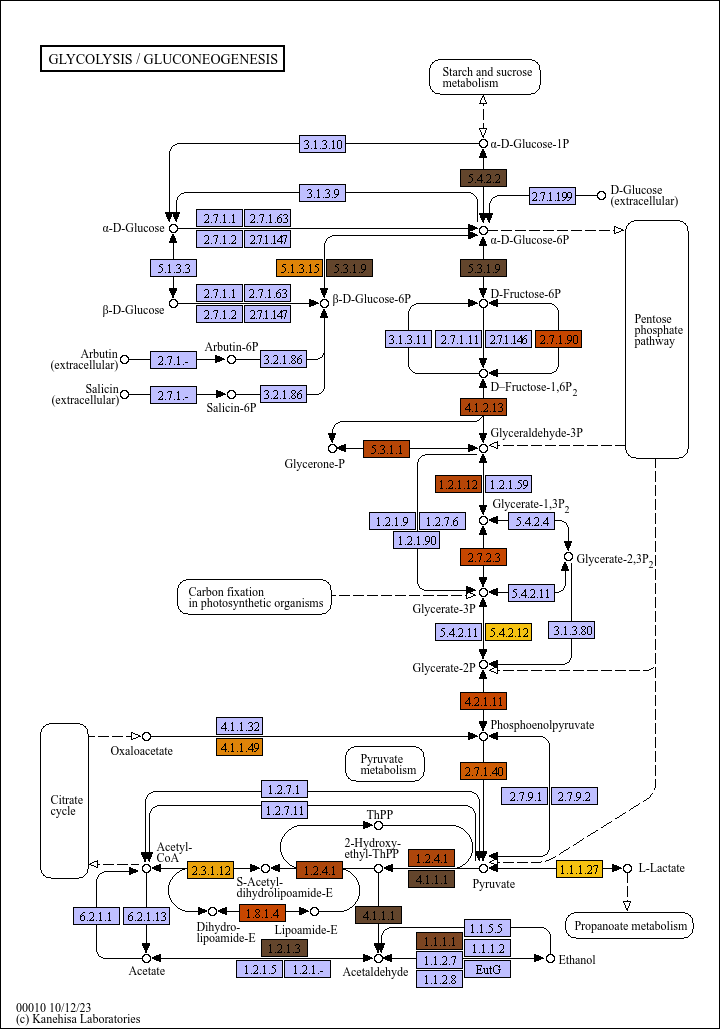

Supplement: Supplementary file 1 [file biomolecules-14-01239-s001.zip › File S2. KEGGpathways/glycolyse_gluconeogenesis_zone.png]

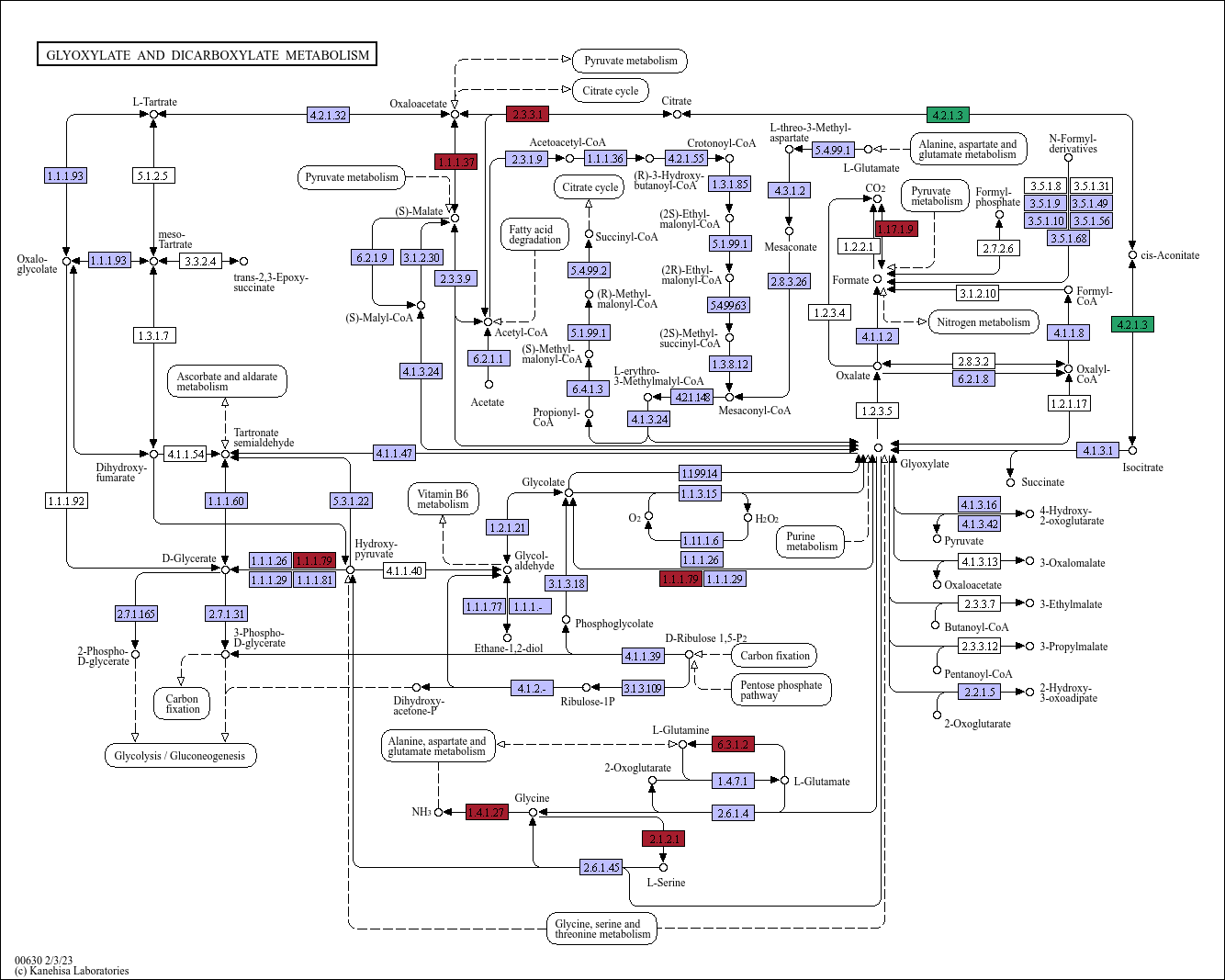

Supplement: Supplementary file 1 [file biomolecules-14-01239-s001.zip › File S2. KEGGpathways/glyoxylate_dicarboxylate_metabo_condition.png]

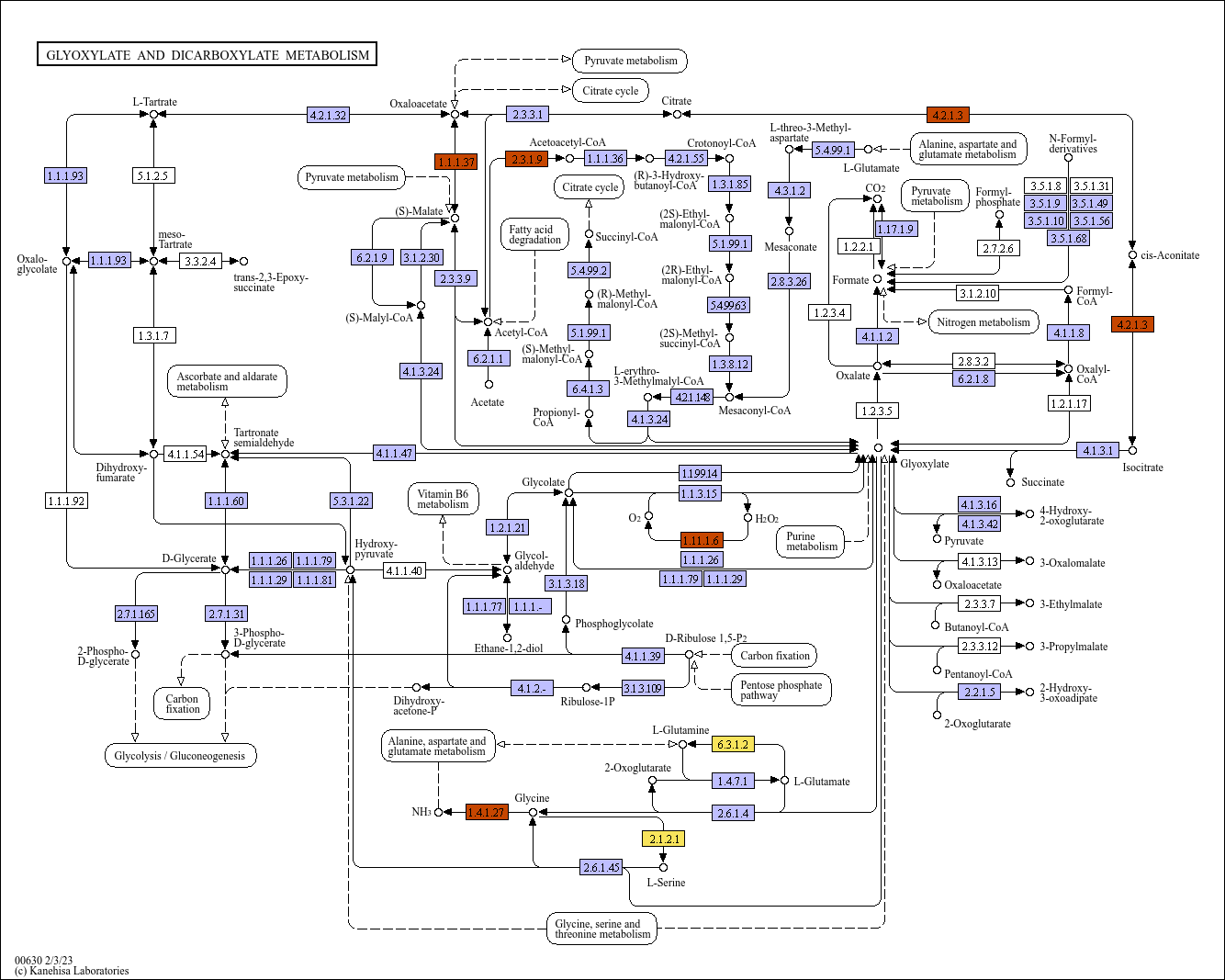

Supplement: Supplementary file 1 [file biomolecules-14-01239-s001.zip › File S2. KEGGpathways/glyoxylate_dicarboxylate_metabo_day.png]

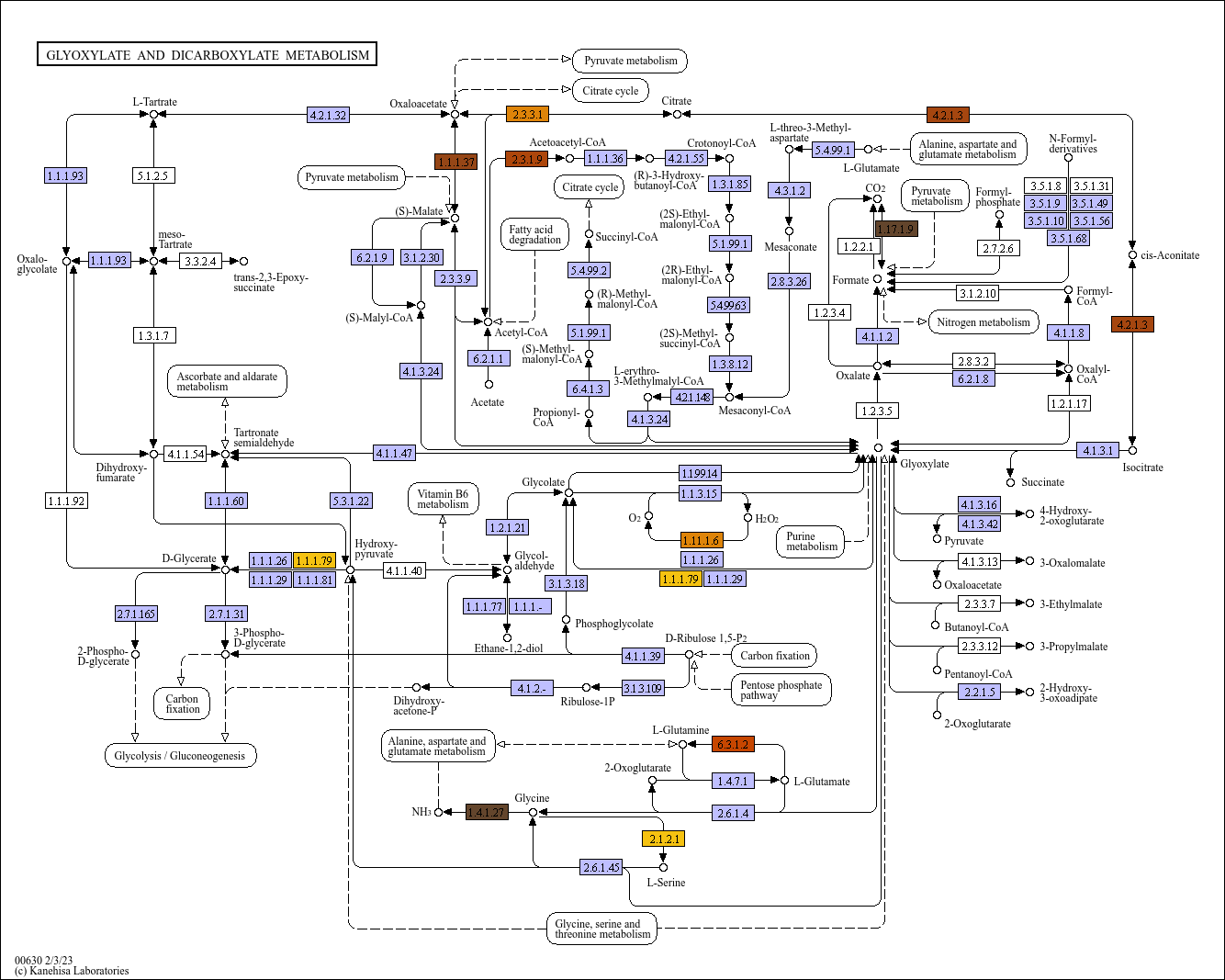

Supplement: Supplementary file 1 [file biomolecules-14-01239-s001.zip › File S2. KEGGpathways/glyoxylate_dicarboxylate_metabo_zone.png]

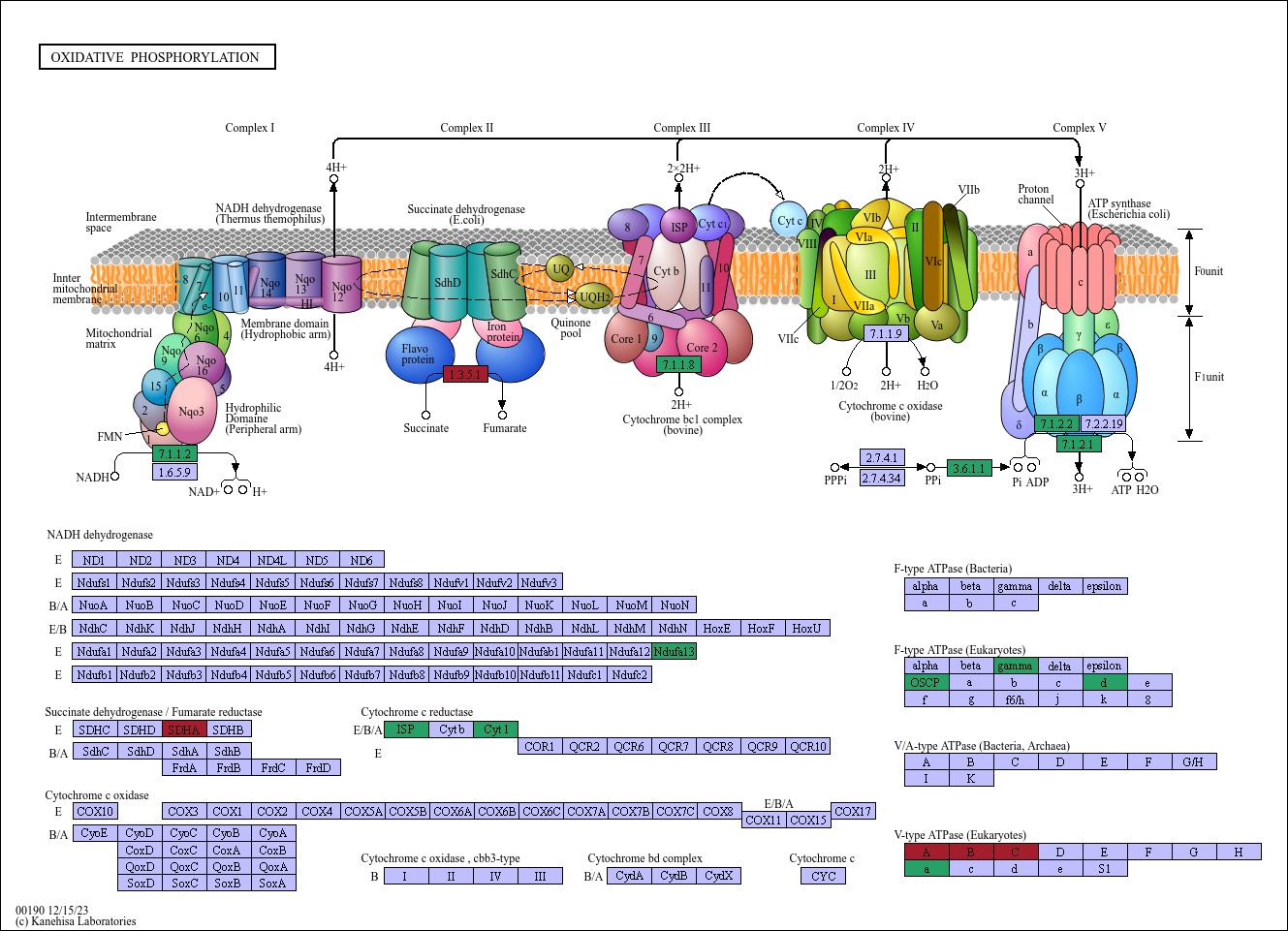

Supplement: Supplementary file 1 [file biomolecules-14-01239-s001.zip › File S2. KEGGpathways/oxydative_phosphorylation_condition.png]

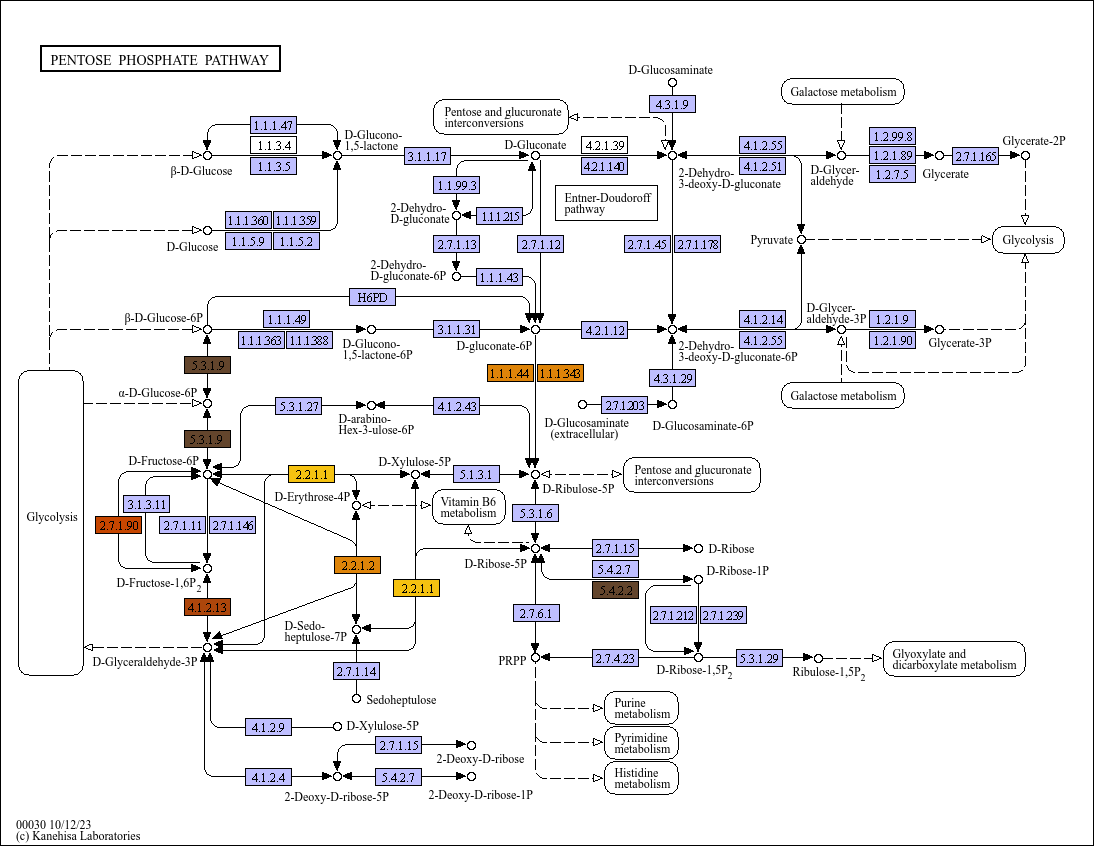

Supplement: Supplementary file 1 [file biomolecules-14-01239-s001.zip › File S2. KEGGpathways/pentose_phosphate_zone.png]

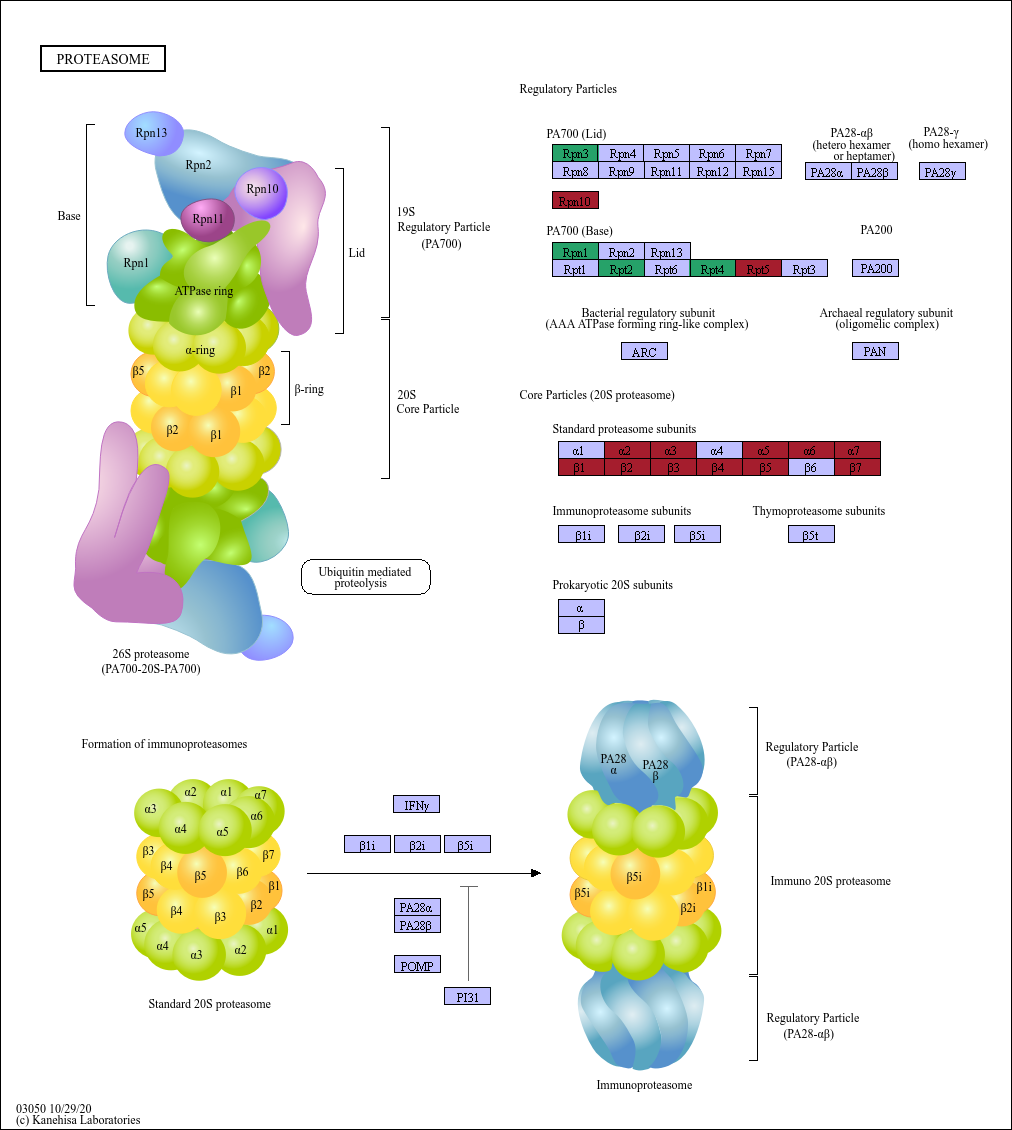

Supplement: Supplementary file 1 [file biomolecules-14-01239-s001.zip › File S2. KEGGpathways/proteasome_condition.png]

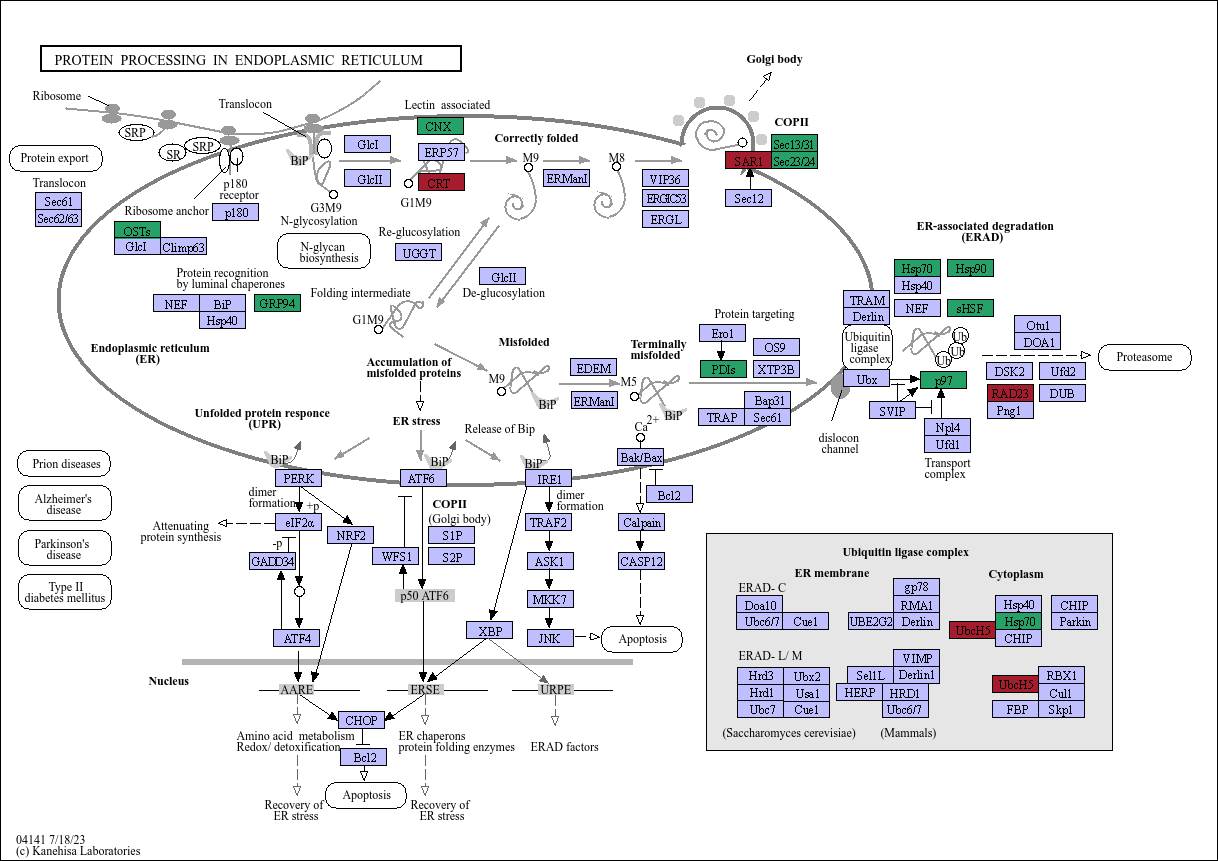

Supplement: Supplementary file 1 [file biomolecules-14-01239-s001.zip › File S2. KEGGpathways/protein_processing_RE_condition.png]

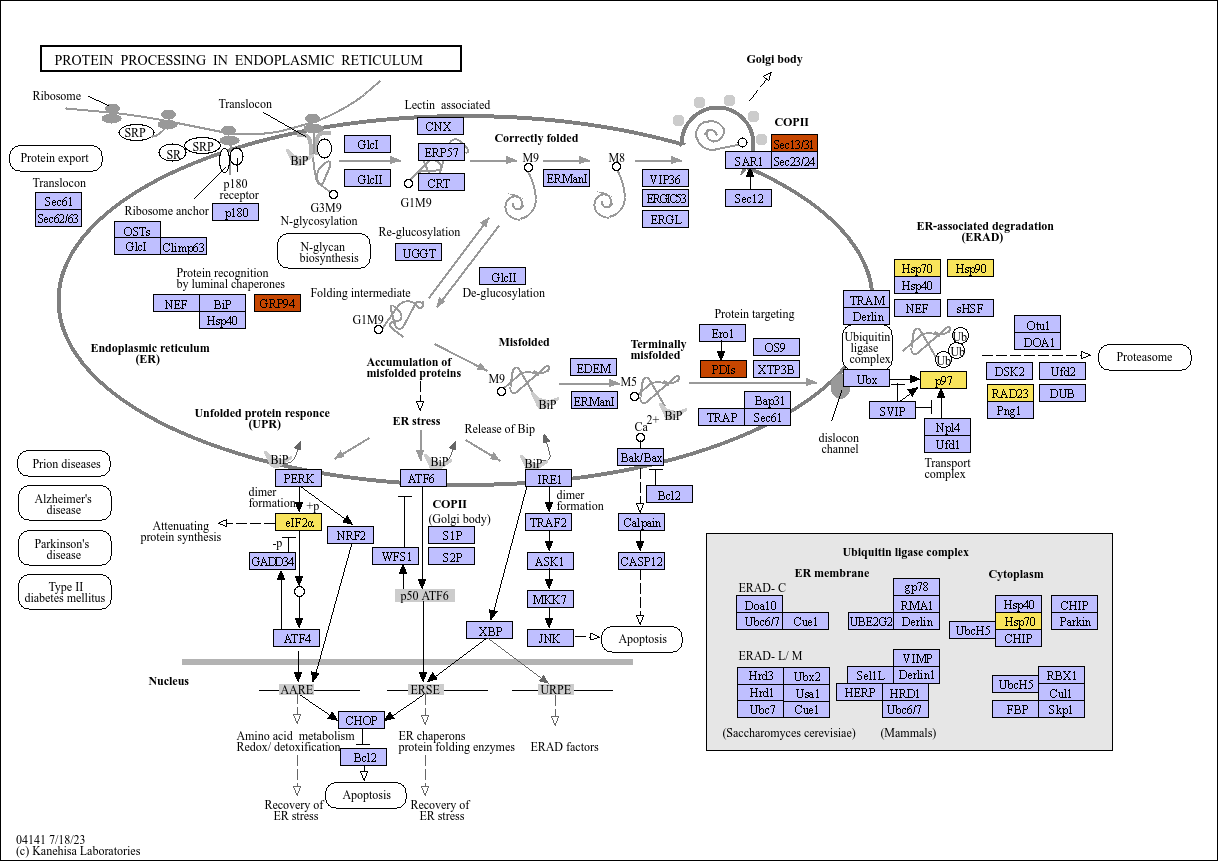

Supplement: Supplementary file 1 [file biomolecules-14-01239-s001.zip › File S2. KEGGpathways/protein_processing_RE_day.png]

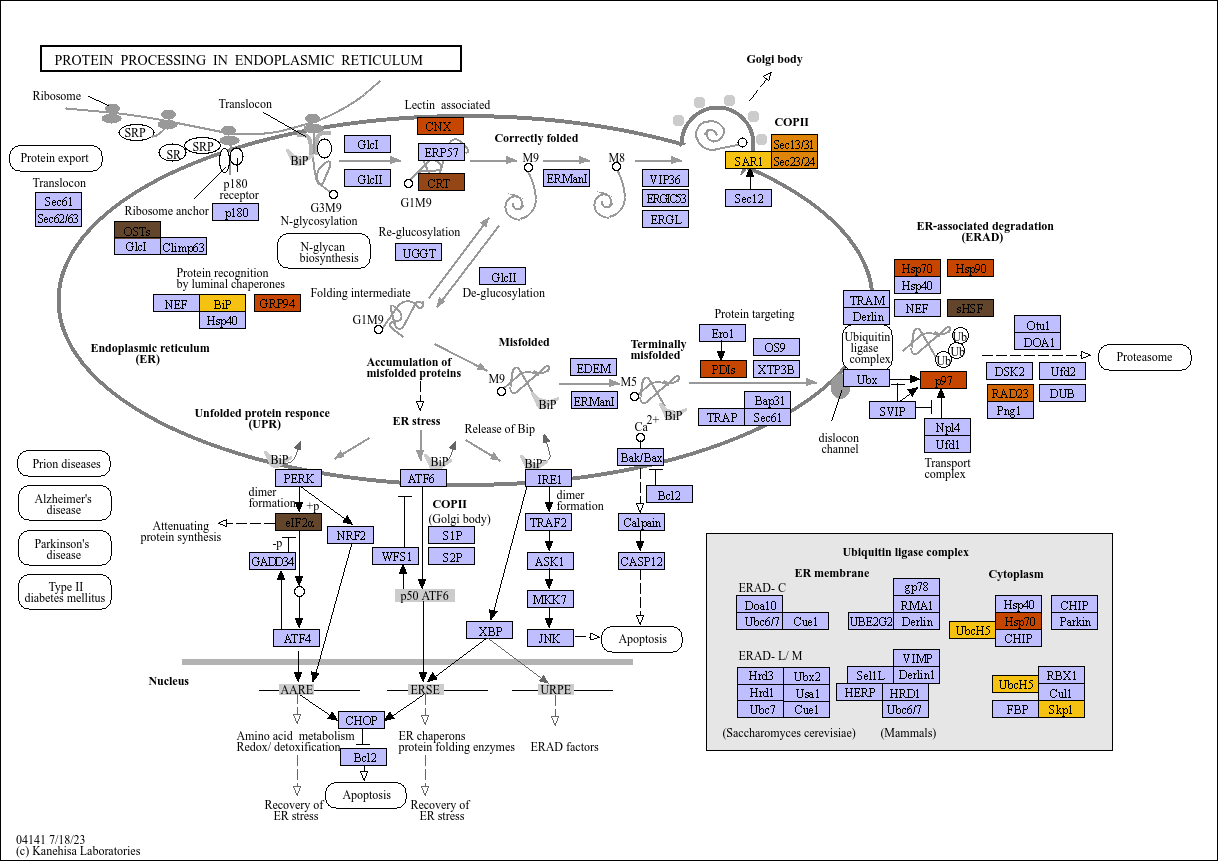

Supplement: Supplementary file 1 [file biomolecules-14-01239-s001.zip › File S2. KEGGpathways/protein_processing_RE_zone.png]

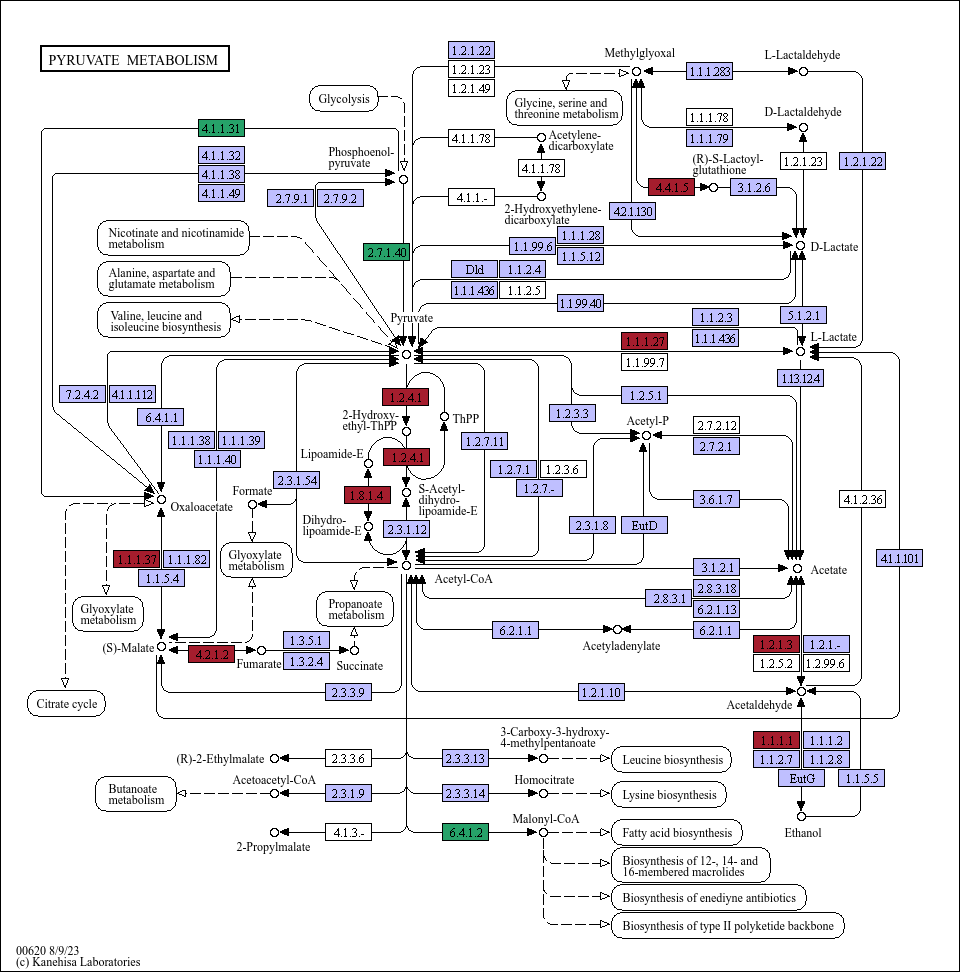

Supplement: Supplementary file 1 [file biomolecules-14-01239-s001.zip › File S2. KEGGpathways/pyruvate_metabolism_condition.png]

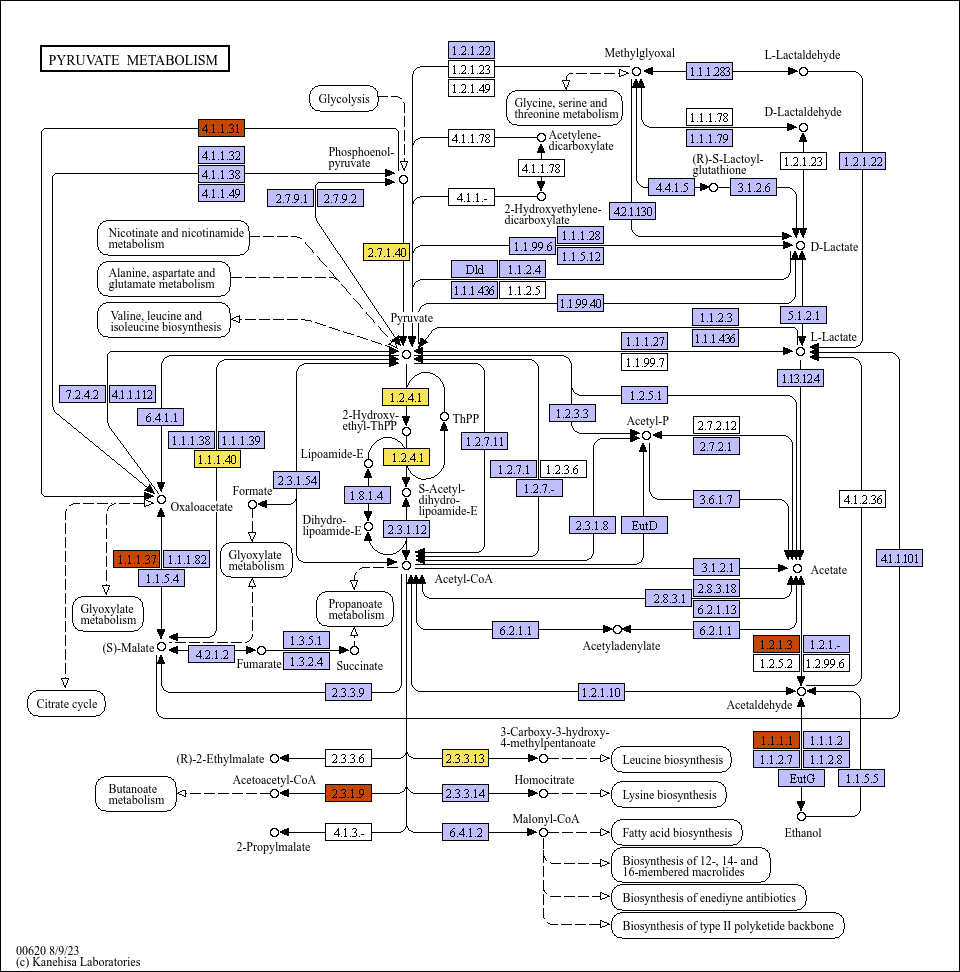

Supplement: Supplementary file 1 [file biomolecules-14-01239-s001.zip › File S2. KEGGpathways/pyruvate_metabolism_day.png]

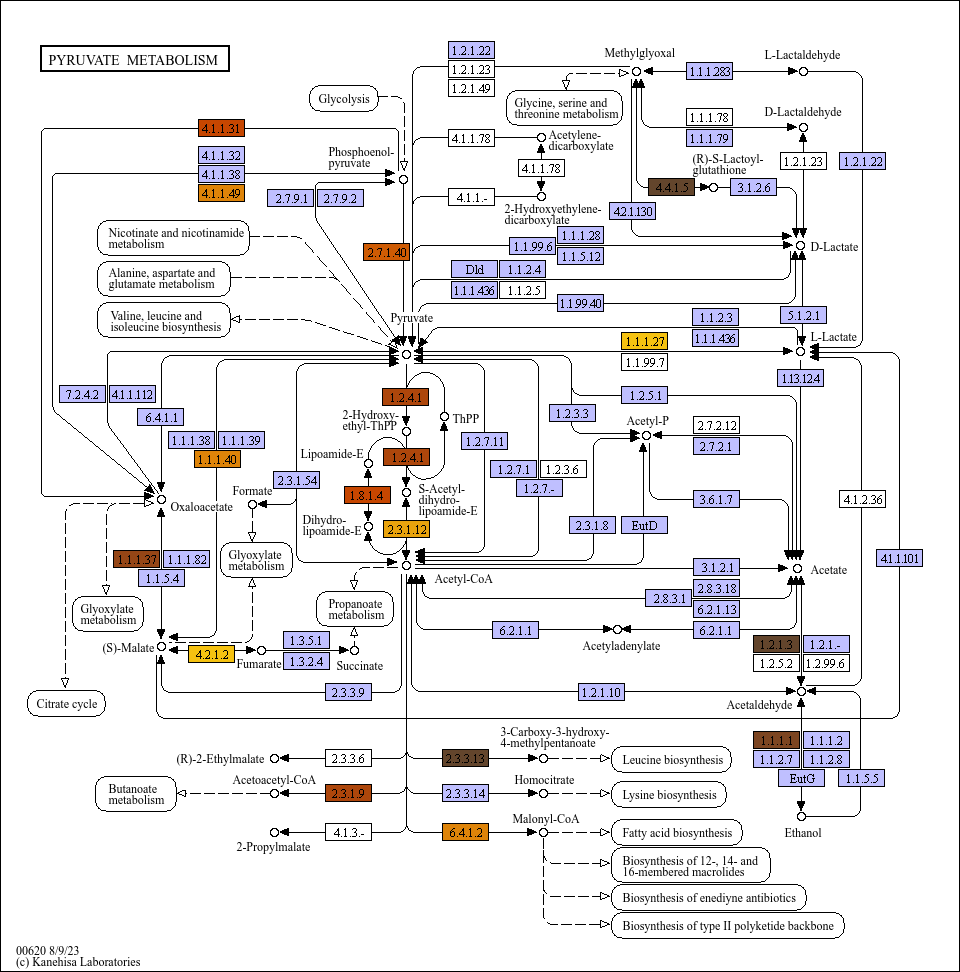

Supplement: Supplementary file 1 [file biomolecules-14-01239-s001.zip › File S2. KEGGpathways/pyruvate_metabolism_zone.png]

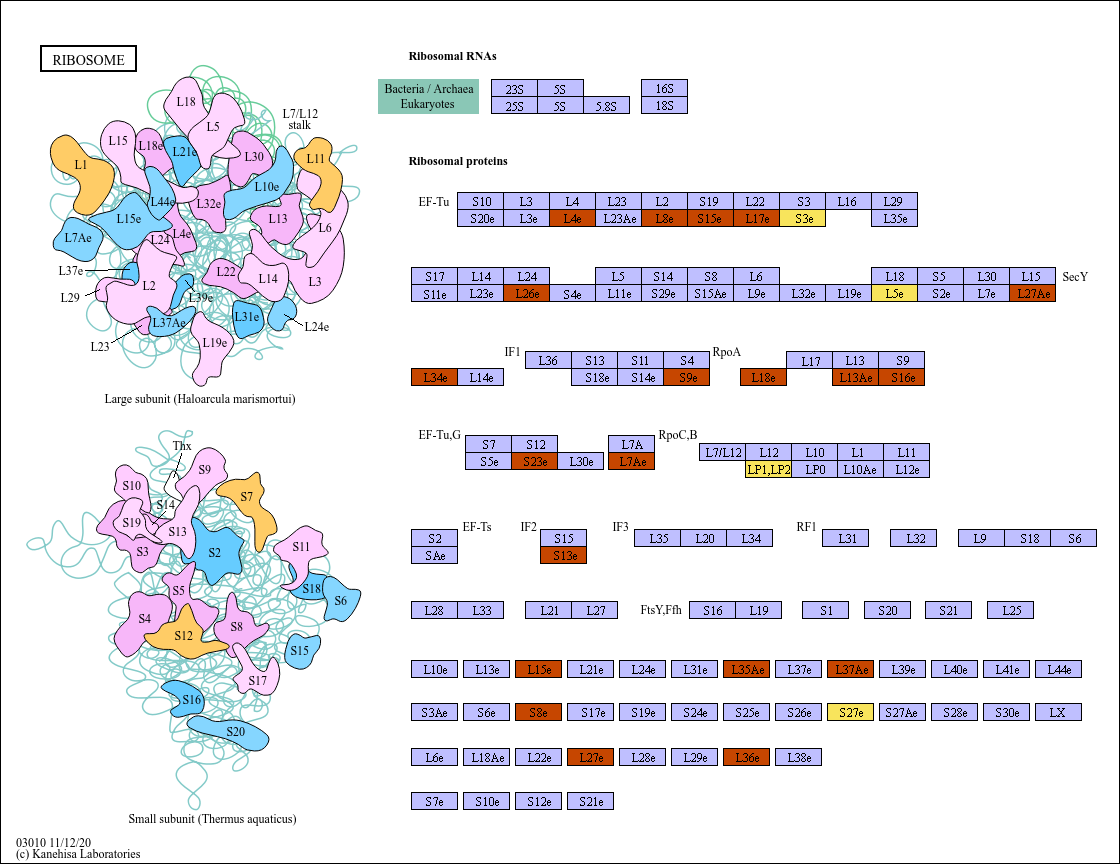

Supplement: Supplementary file 1 [file biomolecules-14-01239-s001.zip › File S2. KEGGpathways/ribosome_day.png]

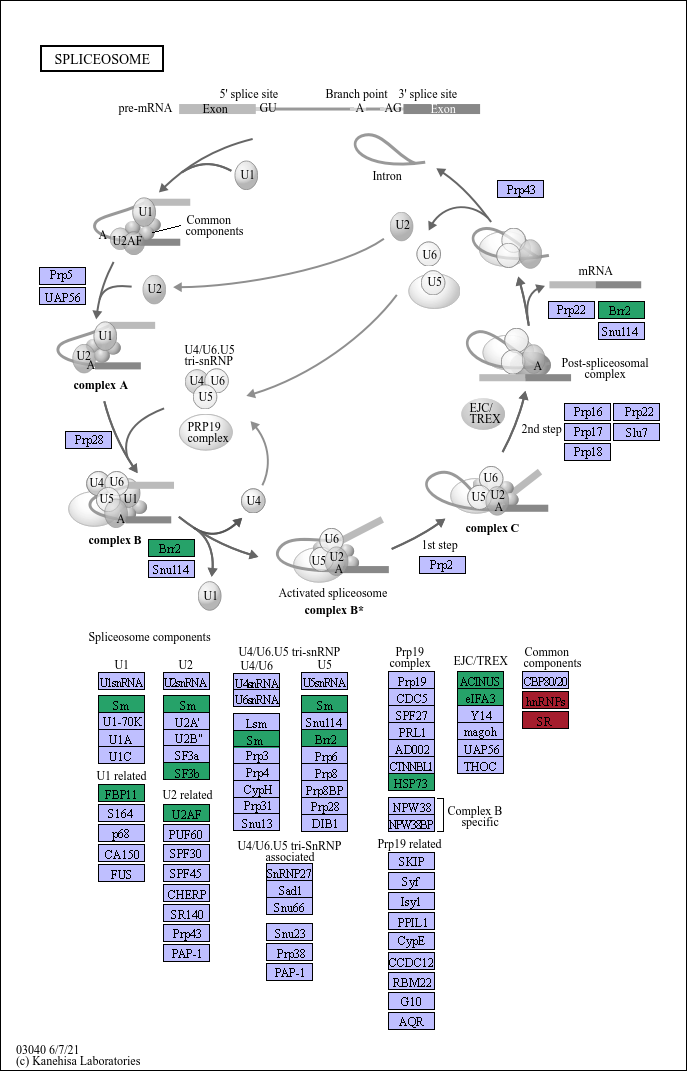

Supplement: Supplementary file 1 [file biomolecules-14-01239-s001.zip › File S2. KEGGpathways/spiceosome_condition.png]

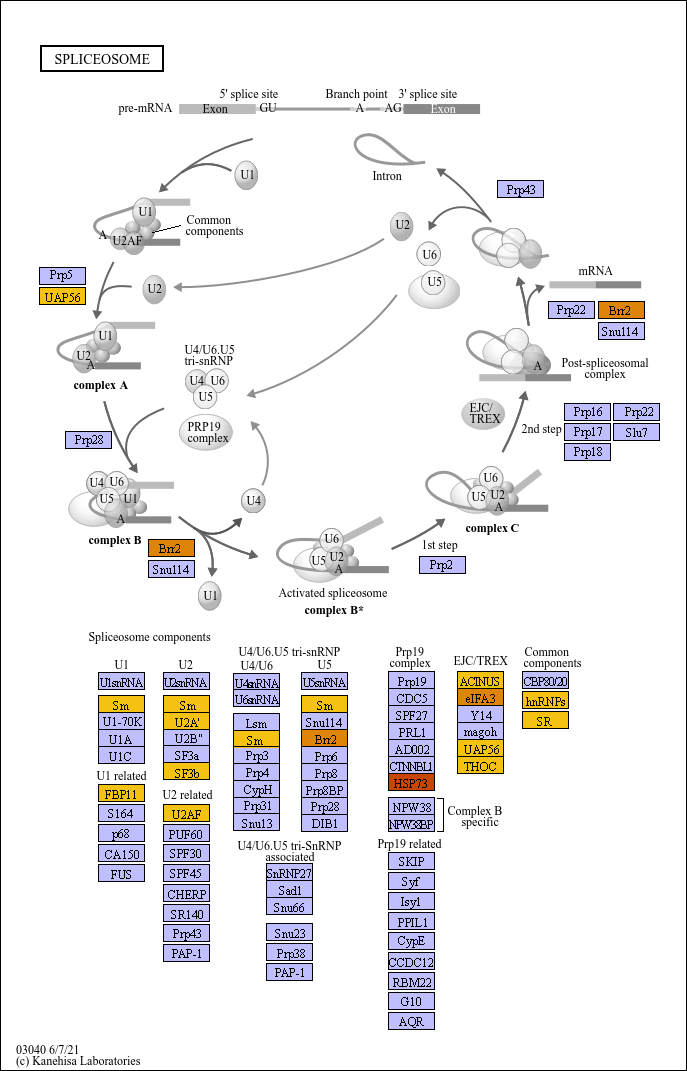

Supplement: Supplementary file 1 [file biomolecules-14-01239-s001.zip › File S2. KEGGpathways/spiceosome_zone.png]

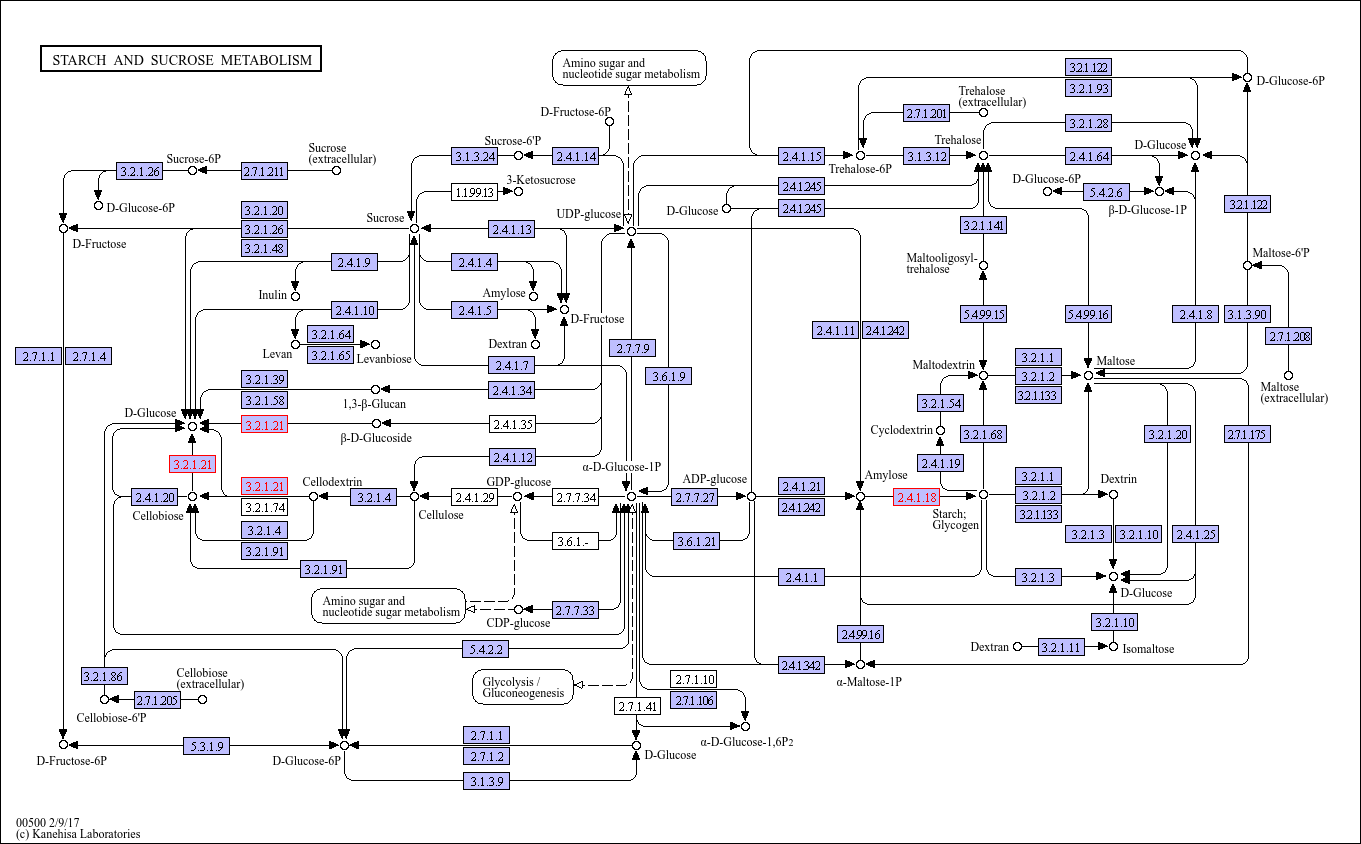

Supplement: Supplementary file 1 [file biomolecules-14-01239-s001.zip › File S2. KEGGpathways/starch_glucose_cjI.png]

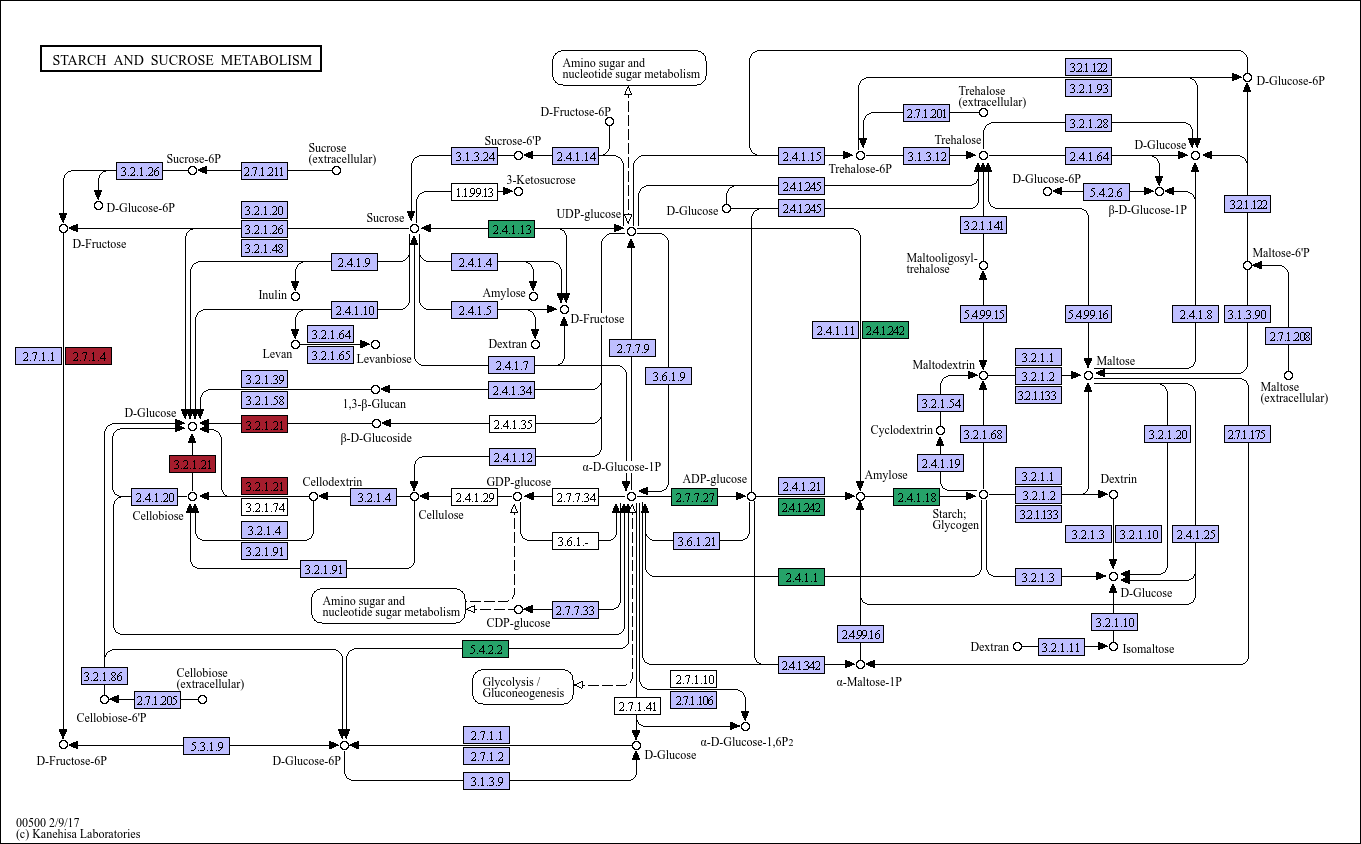

Supplement: Supplementary file 1 [file biomolecules-14-01239-s001.zip › File S2. KEGGpathways/starch_glucose_condition.png]

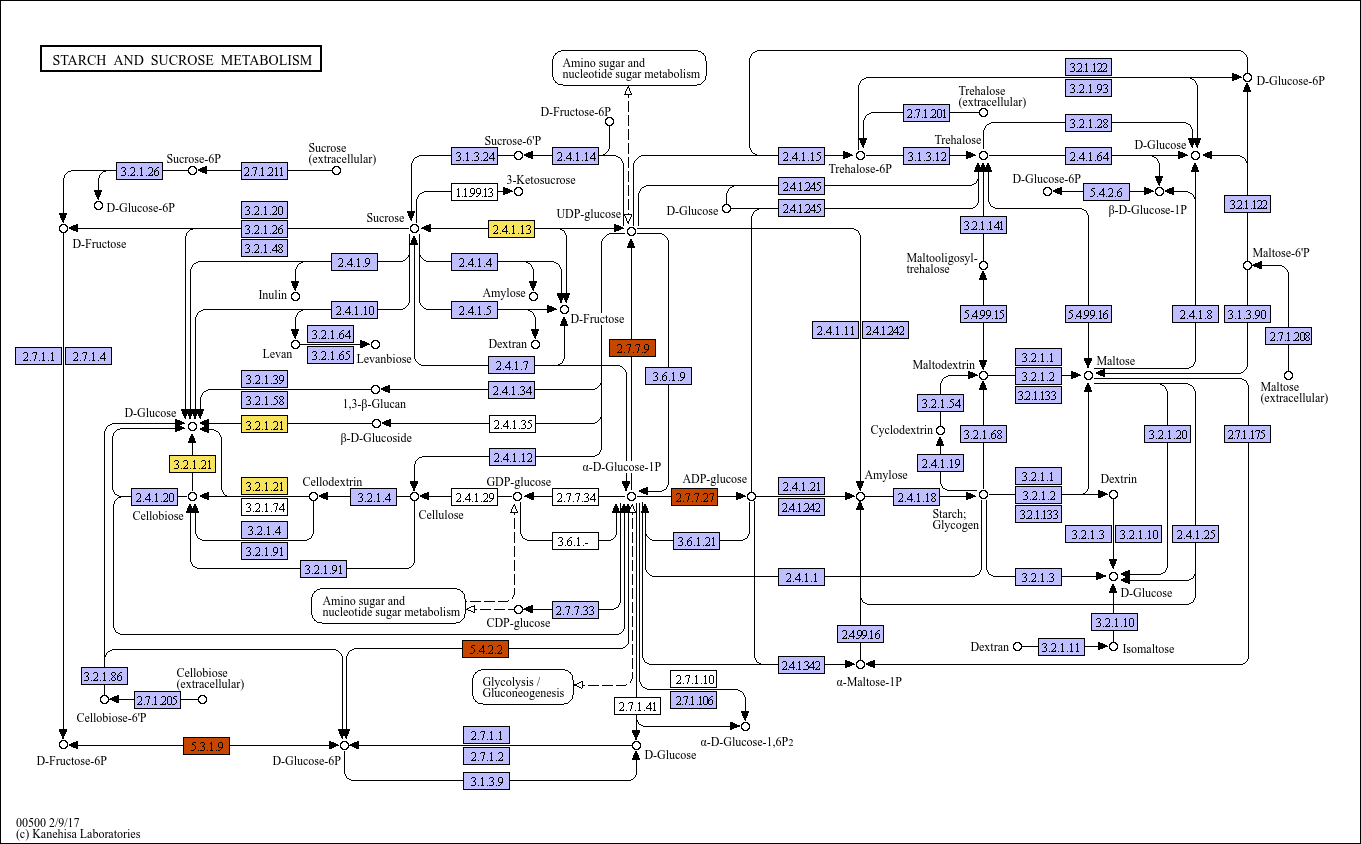

Supplement: Supplementary file 1 [file biomolecules-14-01239-s001.zip › File S2. KEGGpathways/starch_glucose_day.png]

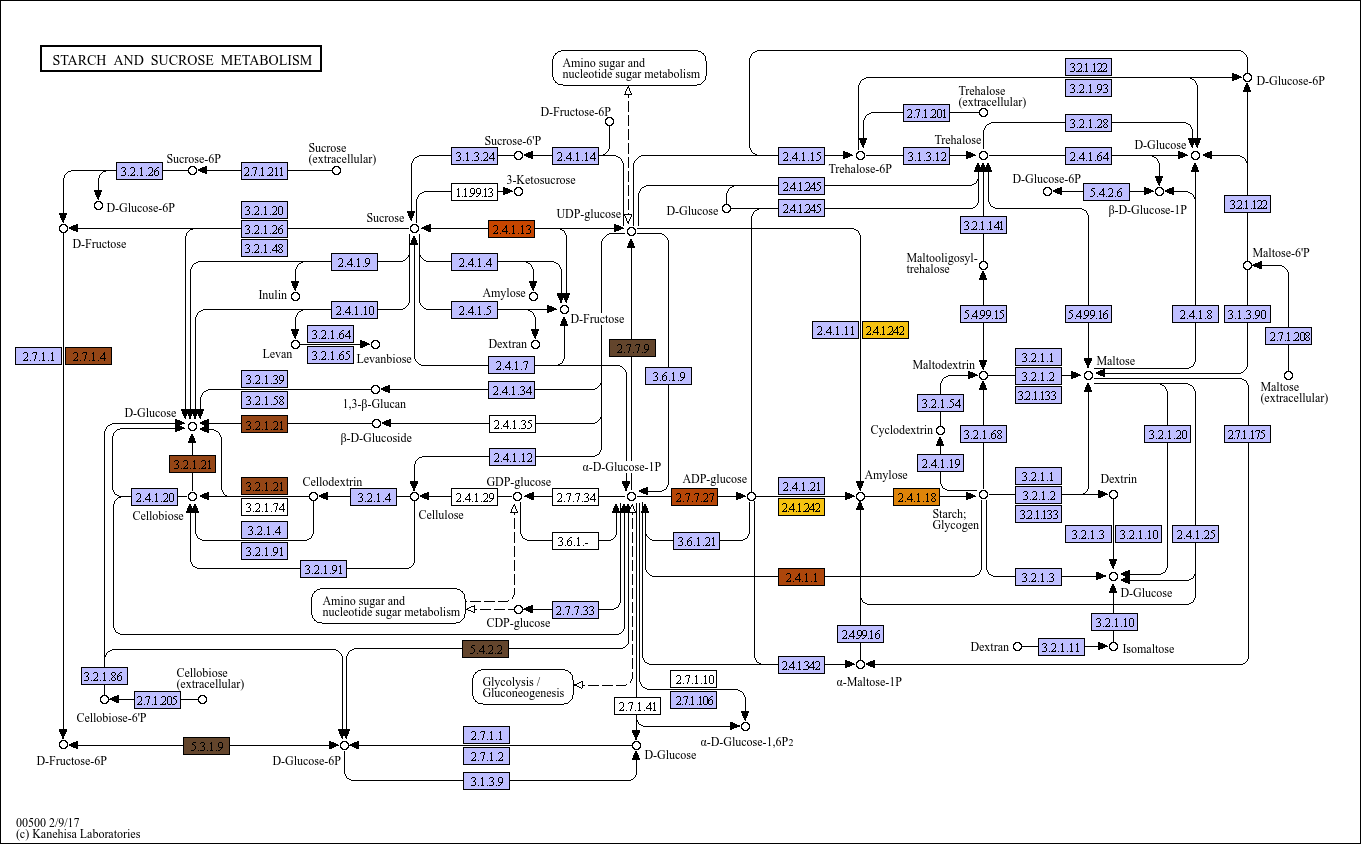

Supplement: Supplementary file 1 [file biomolecules-14-01239-s001.zip › File S2. KEGGpathways/starch_glucose_zone.png]

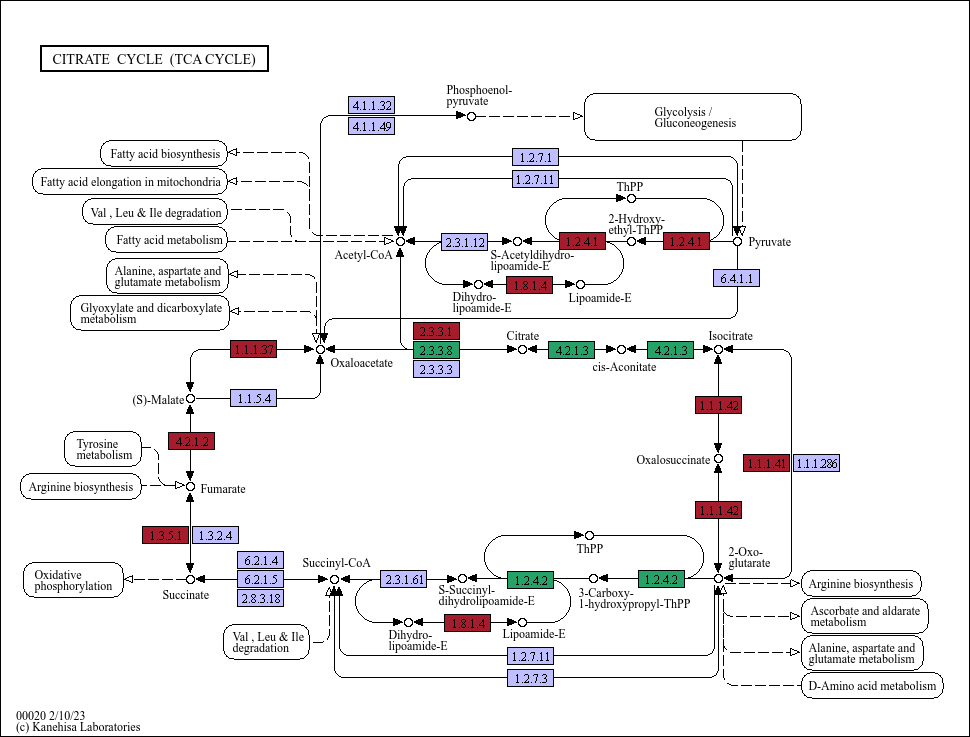

Supplement: Supplementary file 1 [file biomolecules-14-01239-s001.zip › File S2. KEGGpathways/TCA_condition.png]

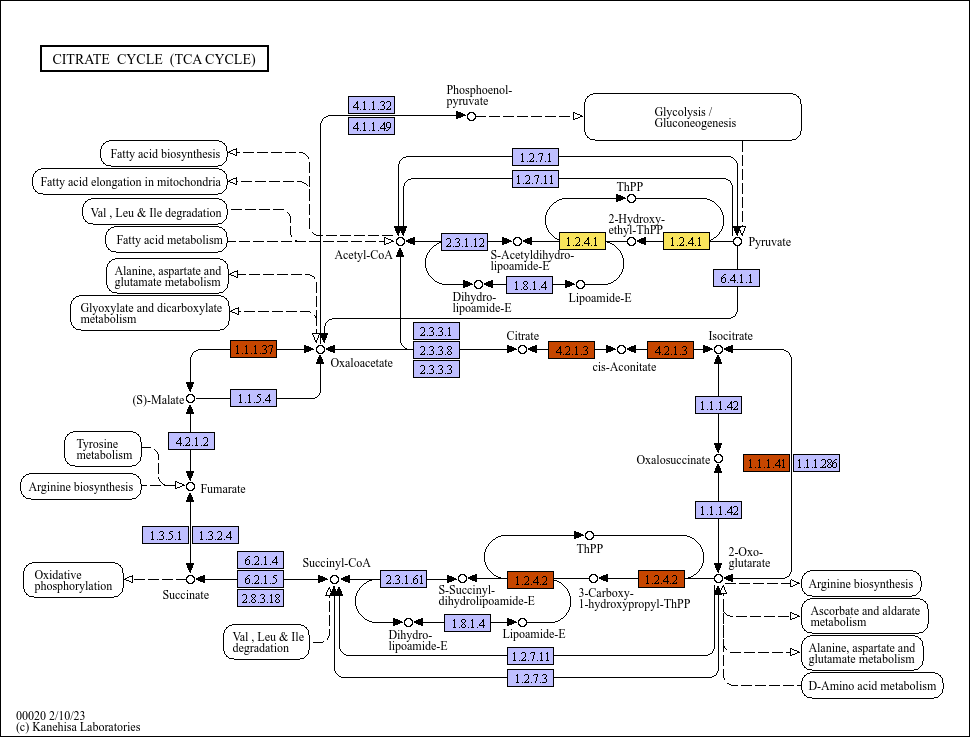

Supplement: Supplementary file 1 [file biomolecules-14-01239-s001.zip › File S2. KEGGpathways/TCA_day.png]

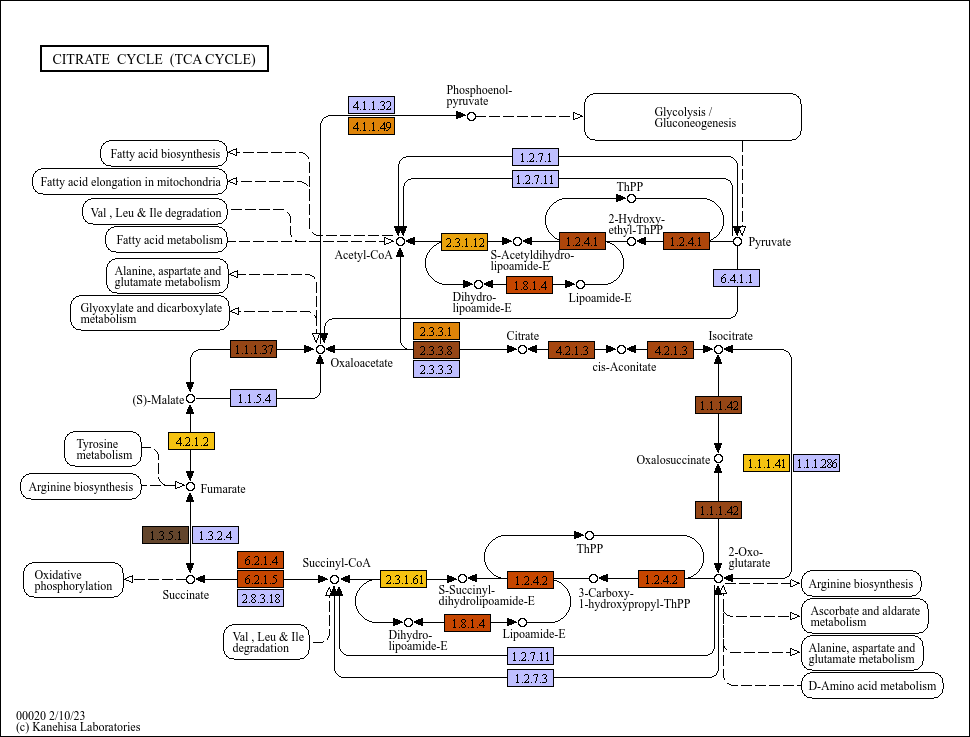

Supplement: Supplementary file 1 [file biomolecules-14-01239-s001.zip › File S2. KEGGpathways/TCA_zone.png]
